# Donor-Recipient Size Mismatch is a Critical Determinant of Kidney Transplant Outcome

**DOI:** 10.1016/j.ekir.2026.107100

**Published:** 2026-09-23

**Authors:** Dharesh Raj Amarnath, Georgios Kourounis, Emily K. Glover, Mohammed Zeeshan Akhtar, Neil S. Sheerin, Colin H. Wilson, Samuel J. Tingle

**Affiliations:** 1 Newcastle University School of Medicine, Newcastle upon Tyne, UK; 2 National Institute of Health and Care Research Blood and Transplant Research Unit, Newcastle University and Cambridge University, Newcastle upon Tyne, UK; 3 Institute of Transplantation, Freeman Hospital, Newcastle upon Tyne, UK; 4 Translational and Clinical Research Institute, Newcastle University, Newcastle upon Tyne, UK; 5 Renal Services, Newcastle upon Tyne Hospitals NHS Foundation Trust, Newcastle upon Tyne, UK; 6 Recanati/Miller Transplantation Institute, Mount Sinai Hospital, New York, USA

**Keywords:** body surface area (BSA) ratio, donor-recipient size mismatch, kidney transplantation, OPTN database

## Abstract

**Introduction:**

In kidney transplantation, the donor kidney should provide sufficient nephron mass to meet recipient demands. Although nephron mass cannot be easily measured, size-mismatch metrics could be useful surrogates for nephron supply and requirement. However, the impact of donor-recipient size-mismatch on post-transplant-kidney outcome remains uncertain.

**Methods:**

Population cohort study using United Network for Organ Sharing data (2010–2024) including adult kidney-only transplants. Multivariable nonlinear (restricted cubic spline) regression models with interaction terms were used to analyze associations. Primary size-mismatch metric was donor-recipient body-surface-area (BSA) ratio, because BSA is a well-established surrogate for nephron number and requirement.

**Results:**

In 155,438 single-kidney transplants, donor-recipient BSA-ratio was associated with 1-year estimated glomerular filtration rate (eGFR), 1- and 5-year graft survival (all *P* < 0.001), with small donor kidneys in larger recipients having consistently inferior outcome. Keeping other factors constant, BSA-ratio in the 5th percentile versus 95th percentile (0.76 vs. 1.37) was associated with a 1-year eGFR of 50.7 versus 71.2 ml/min per 1.73 m^2^ (difference = 20.5, 95% confidence interval [CI] = 19.9–21.1) and the hazard of 5-year graft loss was almost doubled (adjusted hazard ratio [aHR] = 1.93, 95% CI = 1.75–2.13). After donor age, donor-recipient BSA-ratio was most strongly associated with 1-year eGFR, with a stronger association than the other 34 examined factors. Nonlinear interaction analyses revealed the association between BSA-ratio and outcome remained consistent regardless of donor age or donor-recipient sex-mismatch.

**Conclusion:**

Donor-recipient size-mismatch, measured by BSA-ratio, is strongly associated with post-transplant outcome. It is more strongly associated with outcome than most other factors used in current offering schemes and risk indices. Therefore, size-mismatch should be considered in kidney offering schemes and adjusted for as a critical potential covariate and/or confounder in observational studies and prospective trials.

## Graphical abstract

**Figure:**
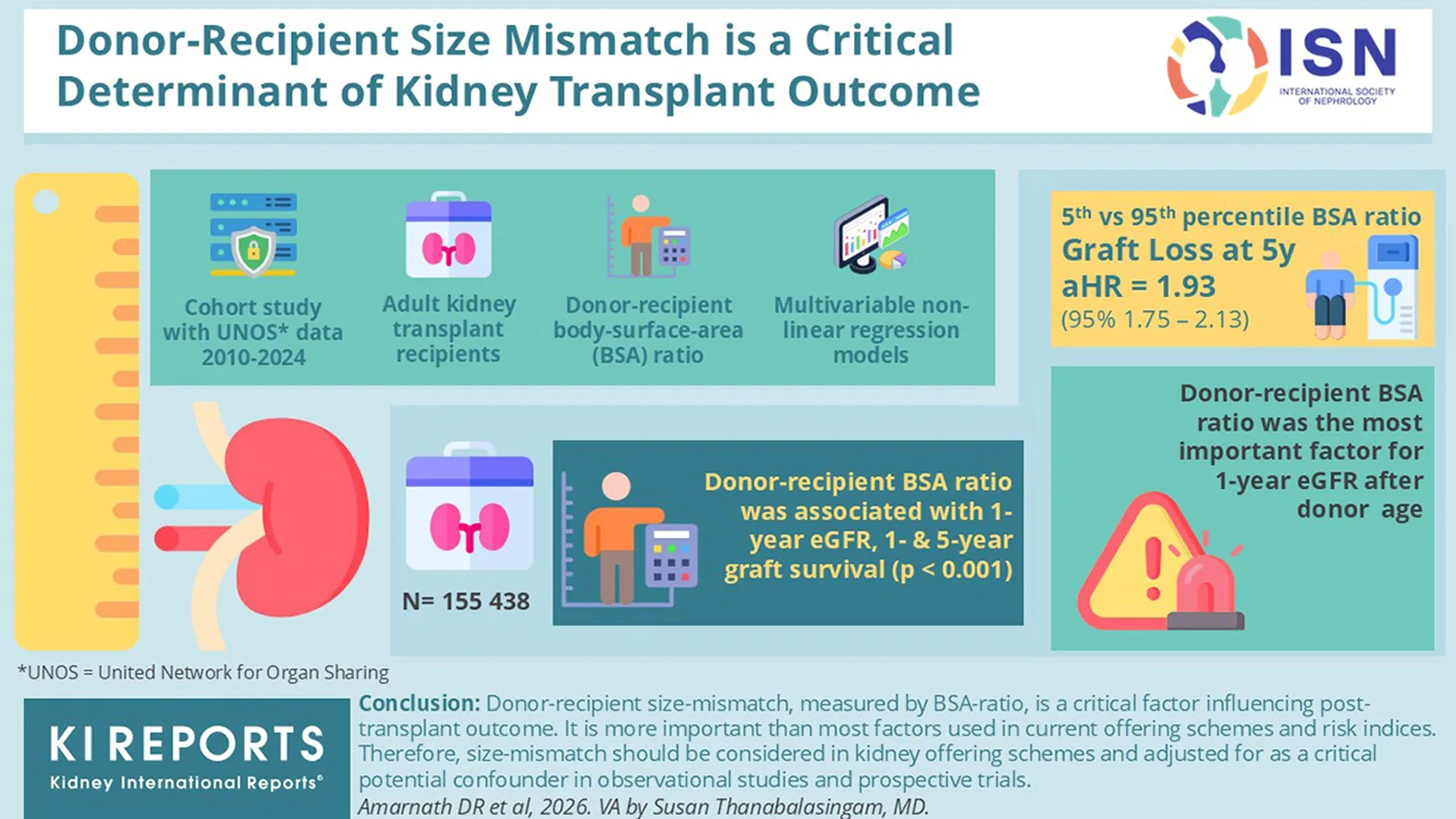

Kidney transplantation offers significant advantages in survival, quality of life, and cost-effectiveness compared with dialysis for patients with end-stage kidney disease.^1^^,^^2^ Optimal donor-recipient matching is essential to improve recipient graft survival, as well as to ensure equity of access. The donor kidney should be able to provide sufficient nephron mass to match the recipient’s demands; a relatively small donor kidney may provide inadequate nephron mass for a large recipient.^3^

Nephron mass is not easily measured directly, but can be estimated using various anthropometric measures such as height, weight, BSA or lean body mass (LBM). BSA is widely considered the best anthropomorphic surrogate measure for functional nephron mass in the absence of kidney disease.^4^ In the context of kidney transplantation, an optimal metric to assess donor-recipient size mismatch would capture the imbalance between the donor available nephron mass (as the numerator) and the recipient nephron mass requirement (as the denominator).

The current literature is conflicting regarding whether donor-recipient size mismatch has an impact on recipient outcomes post kidney transplant. A recent independent review, which included studies over the past 5 decades, highlighted that there is “a critical gap in the transplant literature—despite decades of research and biological plausibility rooted in the nephron dosing hypothesis, there is no consensus on how to define or operationalize size mismatch”.^5^

Although some previous studies have suggested that donor-recipient size mismatch may be associated with post-transplant outcomes,^6^, ^7^, ^8^ they have been limited by analytical approaches that may not fully capture the relationship between donor-recipient size mismatch and outcome, including categorization of continuous size mismatch metrics into arbitrary groups, evaluation of only a single size metric (such as BSA, weight, or height), and in some cases relatively small sample sizes. Furthermore, none have quantified the relative importance of donor-recipient size mismatch compared with established drivers of post-transplant kidney function. The potential implications of donor-recipient size mismatch for equity in kidney offering also remain unexplored. Addressing these gaps is urgently required to identify the impact of size mismatch in kidney transplantation and to optimize kidney offering and recipient outcomes.

In this study, we analyzed various donor-recipient size mismatch measures and their association with kidney transplant outcomes.

## Methods

We performed a population-cohort study using data from the Organ Procurement & Transplantation Network registry, specifically data from STAR and DONORNET supplement files. As data was provided in a fully anonymized format, our institutional review board equivalent determined that study-specific ethical review was not required. We included adult recipients (aged ≥ 18 years at time of transplant) of deceased donor kidney transplants performed between January 1, 2010, and June 30, 2024. Exclusion criteria were uncontrolled donation after circulatory death, donors aged under 18, multiorgan and/or multivisceral transplants, en-bloc pediatric kidney transplants, retransplants, and missing data for donor-recipient BSA ratio. The main cohort was single-kidney transplant recipient, whereas dual-kidney transplant recipients were analyzed separately as an additional exploratory cohort. Data were extracted on June 30, 2025, which was the common closure date of the study, ensuring all patients had a minimum of 1 year post transplant follow-up period.

### Outcomes and Definitions

The primary exposure was donor-recipient BSA ratio, defined as donor BSA divided by recipient BSA. BSA was calculated using Du Bois formula,^9^ based on height and weight at the time of donation or transplant. Secondary exposures included ideal donor-recipient BSA ratio, where donor BSA was defined as the lower value of either actual or ideal body weight (IBW, calculated using Devine formula^10^) to avoid inflation of donor BSA because of excess weight, as excess weight gain as an adult is unlikely to alter nephron mass. In contrast recipient BSA was still calculated using actual height and weight, as excess weight gain (leading to higher BSA) will reflect an increase in nephron requirement.

The ideal donor-recipient BSA ratio was calculated as follows:

$$\text{Ideal}\;\text{donor}-\text{recipient}\;\text{BSA}\;\text{ratio}=\frac{\min\left(\text{Donor}\;\text{actual}\;\text{BSA},\text{Donor}\;\text{ideal}\;\text{BSA}\right)}{\text{recipient}\;\text{actual}\;\text{BSA}}$$

Actual BSA was calculated as follows:

$${\text{BSA}}_{\text{actual}}=0.007184\times \mathrm{weight}\;\mathrm{in}\;kg^{0.425}\times \mathrm{height}\;\mathrm{in}\;cm^{0.725}$$

Ideal BSA was calculated as follows:

$${\text{BSA}}_{\text{ideal}}=0.007184\times {\mathrm{IBW}}^{0.425}\times \mathrm{height}\;\mathrm{in}\;cm^{0.725}$$

IBW was calculated according to sex:

$${\text{IBW}}_{\text{male}}=50+2.3\times \left(0.3937\times \text{height}\;\text{in}\;\text{cm}-60\right)$$

$${\text{IBW}}_{\text{female}}=45.5+2.3\times \left(0.3937\times \text{height}\;\text{in}\;\text{cm}-60\right)$$

Additional secondary exposures included donor height-recipient BSA (defined as donor height divided by recipient BSA, normalized to a median of 1), Non-BSA-normalized donor eGFR (defined as the best donor eGFR [to account for acute kidney injury] calculated using Chronic Kidney Disease Epidemiology Collaboration 2021 formula without ethnicity adjustment,^11^ and multiplied by donor-recipient BSA ratio), donor-recipient height ratio (defined as donor height divided by recipient height), donor-recipient weight ratio (defined as donor weight divided by recipient weight) and donor-recipient LBM ratio (defined as calculated donor BSA by recipient BSA, using Boer formula^12^).

Primary outcome of interest was recipient 1-year eGFR (using Chronic Kidney Disease Epidemiology Collaboration 2021 formula^11^), with recipients losing their graft before 1 year given a nominal eGFR value of 10 ml/min per 1.73 m^2^.^13^ This was selected as a surrogate of long-term graft survival.^14^ Secondary outcomes were death-censored graft survival (censored at 1 year and 5 years) and delayed graft function (defined as the need for dialysis in the first week after transplant). As a sensitivity analysis, overall graft survival (without censoring for death at 1 year and 5 years) was assessed.

### Statistical Analysis

We used the same statistical approach as described previously.^13^ Multiple imputation was performed by generating 20 imputed data sets to account for missing data, using predictive mean matching with bootstraps to build rich additive restricted cubic spline models (aregImpute; Hmisc R package).^15^, ^16^, ^17^ This approach preserves nonlinear relationships. In the imputation models, we included outcome variables to preserve their relationship with missing covariates.^18^ For time-to-event outcomes, we included the event indicator variable and cumulative hazard of the event.^18^

Multivariable linear regression model was used to analyze the association of donor-recipient size mismatch with recipient 1-year eGFR. Multivariable Cox regression was performed to assess graft survival. Multivariable logistic regression was performed to assess the association with delayed graft function. Results were pooled from 20 imputed data sets using Rubin’s rules,^19^ adjusting for a wide range of covariates. The full list of covariates is provided in Supplementary Table S1. Potential covariates were selected based on previous research and clinical expertise; statistical variable selection techniques (e.g., stepwise selection), or removal of covariates after seeing the regression model results, were avoided.^20^ A single prespecified set of covariates was used for all analyses. Donor and recipient sex were defined according to the variables recorded in the OPTN registry. When analyzing the association of donor-recipient size mismatch factors, we also adjusted for the corresponding donor variable to identify the independent association of mismatch. This was critical in assessing whether mismatch was independently associated with post-transplant outcome, versus kidneys from larger donors simply having improved outcome regardless of recipient size.

We employed hierarchical modelling to adjust for transplant center as a determinant of post-transplant outcome, using a mixed-effects logistic regression model with random intercepts for transplant center.^21^ To adjust the model variance for clustering to account for individual donors donating kidneys to separate recipients, we used the Huber-White Sandwich methods via the rms robcov function to generate cluster robust standard errors.^22^

Restricted cubic splines with 4 knots (5th, 35th, 65th, and 95th percentiles) were used to analyze continuous variables to avoid the assumption of nonlinear relationships. Additional models were built allowing for interactions, to assess whether the various donor-recipient mismatch exposures were inaccurate based on specific donor, recipient, or transplant factors.

We performed a simple simulation analysis to assess how accounting for donor-recipient size mismatch in offering schemes could improve equity. Two hypothetical recipients were defined, with height and weight set at the 5th and 95th percentiles to represent small and large recipients respectively. All other recipient factors were set at their cohort-wide average values. The potential donor pool for each recipient was then defined to simulate the following 3 offering scheme scenarios: only donors with ideal BSA within 10% of recipient BSA, donors with ideal BSA within 20% of recipient BSA, and a final policy where recipients could have been matched to any donor. For each offering scheme, we performed bootstrapping sampling of 100,000 donors for each of the 2 hypothetical recipients. Predicted 1-year eGFR was calculated using the multivariable regression model described above.

Kaplan-Meier plots were generated to show crude graft survival, stratified by donor-recipient BSA ratio. Continuous variables are given as median and interquartile range. Outputs of models are given as effect estimates with 95% CIs. All analyses were performed in R (R Foundation for Statistical Computing, Vienna, Austria),^23^ using the following packages: tidyverse, rms, rmsMD, lem4, Hmisc and survminer.^15^^,^^21^^,^^24^, ^25^, ^26^, ^27^ Regression results tables and plots were generated using the rmsMD package.^28^

The relative importance of donor-recipient BSA ratio compared with other variables included in the multivariable models was assessed using likelihood ratio chi-square values for survival models (1- and 5-year graft survival outcomes), and the equivalent partial F-tests for ordinary least squares model (1-year eGFR outcome).^29^ Sensitivity analyses were performed using Wald chi-square statistics. Variables were ranked based on the magnitude of the corresponding test statistic.

## Results

We included 156,917 kidney recipients from January 1, 2010, to June 30, 2024. Of these, 155,438 were single-kidney transplant recipients, whereas 1479 were dual-kidney transplant recipients. The single-kidney transplant recipients (*n* = 155,438) were kept as the main cohort, whereas dual-kidney transplant recipients (*n* = 1479) were analyzed separately as an additional exploratory cohort. Information on cohort selection is given in our study flow diagram (Figure 1). Key cohort demographics are given in Table 1, with additional demographics and description of missing data in Supplementary Table S2 and Supplementary Figure S1 (with additional definitions in Supplementary Table S3). The median (IQR) donor-recipient BSA ratio was 1.01 (IQR 0.90–1.15). The 5th and the 95th percentiles for donor-recipient BSA ratio were 0.761 (e.g., donor: 167 cm, 70 kg; recipient: 188 cm, 109 kg) and 1.372 (e.g., donor: 188 cm, 98 kg; recipient: 163 cm, 58 kg) respectively, with 7772 recipients having BSA ratios below the 5th or above the 95th percentile. The median (IQR) ideal donor-recipient BSA ratio was 0.91 (IQR 0.81–1.02). The distribution of these BSA ratios is shown in Figure 2a and b.

**Figure 1:**
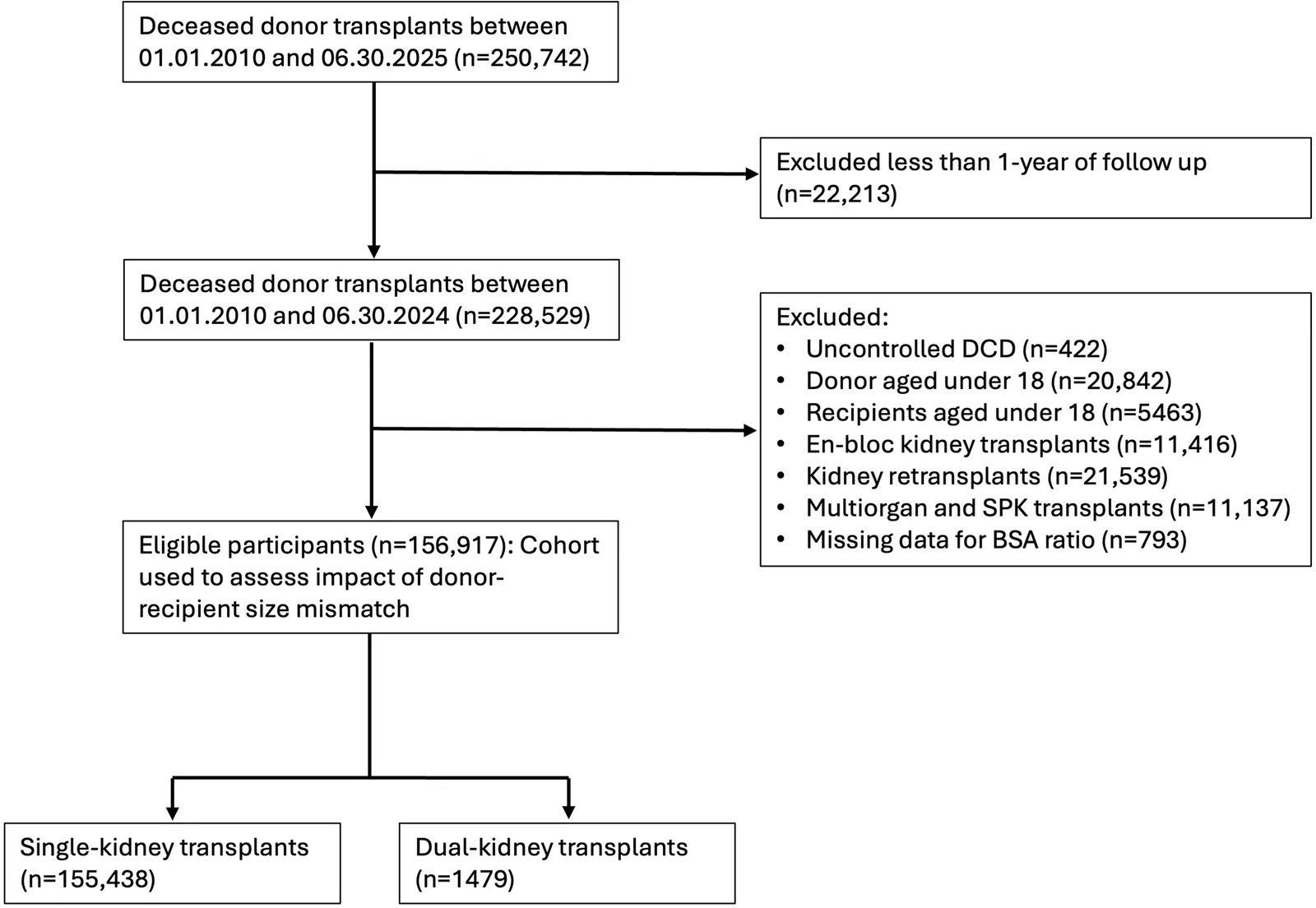
Flowchart and study cohorts. BSA, body surface area; DCD, donation after circulatory death; SPK, simultaneous pancreas-kidney transplant.

**Table 1:** Cohort demographics

Variables	Overall (*N* = 156,917)
Donor age (yrs)	43.0 (31.0–53.0)
Donor BSA (m^2^)	1.96 (1.80–2.13)
Cause of death	
Head trauma	42,683 (27.2%)
Cerebrovascular/stroke	42,272 (26.9%)
Anoxia with PDCA	33,048 (21.1%)
Drug overdose	23,954 (15.3%)
Other	10,542 (6.7%)
Donor type	
DBD	114,954 (73.3%)
DCD-NRP	1891 (1.2%)
DCD-SRR	37,657 (24.0%)
DCD-unknown	2415 (1.5%)
Donor diabetes history	
No	140,927 (89.8%)
Yes	142,63 (9.1%)
Donor peak creatinine (mg/dl)	1.40 (1.06–1.93)
Recipient age (yrs)	57.0 (46.0–65.0)
Recipient BSA (m^2^)	1.94 (1.77–2.11)
Donor-recipient sex match	
Donor male à recipient male	59,619 (38.0%)
Donor female à recipient male	35,926 (22.9%)
Donor male à recipient female	37,064 (23.6%)
Donor female à recipient female	24,308 (15.5%)
Cold Ischemic time (h)	18.0 (12.7–23.2)

BSA, body surface area; DBD, donation after brain death; DCD, donation after circulatory death; NRP, normothermic regional perfusion; PDCA, pre-donation cardiac arrest; SRR, super-rapid recovery.

Continuous variables are reported as median (IQR).

**Figure 2:**
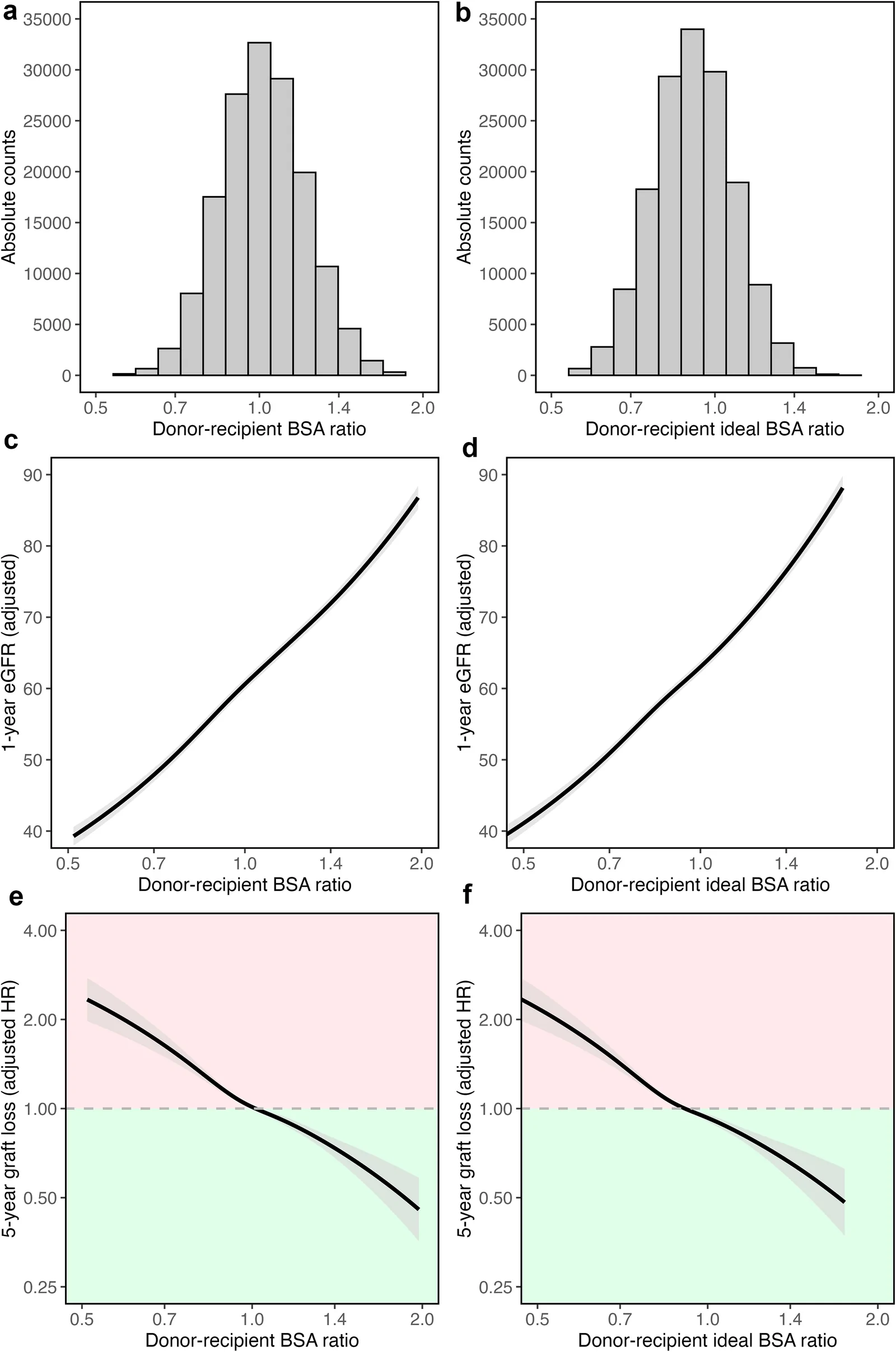
Distribution of donor-recipient BSA ratios and their association with outcome. Histogram of distribution of (a) donor-recipient BSA ratio and (b) ideal donor-recipient BSA ratio The x-axis is log-scaled; each bar represents a 10% increase in BSA ratio (e.g., 0.50-0.55, 0.55-0.61, etc). The y-axis represents absolute counts of patients in each bin. Association between (c) donor-recipient BSA ratio and (d) ideal donor-recipient BSA ratio on 1-year eGFR. Association between (e) donor-recipient BSA ratio and (f) ideal donor-recipient ratio on 5-year graft loss. Grey lines represent the adjusted change in outcome, with grey shaded areas representing the 95% confidence intervals. The green region represents superior outcome compared with the reference, whereas the red region signifies inferior outcome. BSA, body surface area; eGFR, estimated glomerular filtration rate; HR, heart rate.

### Donor-Recipient BSA Ratio as a Measure of Size Mismatch

A multivariable linear regression model was used to analyze the association of donor-recipient BSA ratio with recipient 1-year eGFR, adjusting for a wide range of factors (Figure 2, full model details in Supplementary Table S4 and Supplementary Figure S2). Donor-recipient BSA ratio was significantly associated with 1-year eGFR (*P* < 0.001). As seen in Figure 2c, kidneys from small donors into larger recipients consistently showed inferior outcome. Every 0.1 increase in donor-recipient BSA ratio (e.g., an increase from 1 to 1.1 reflects 10% higher BSA in the donor than recipient) was associated with a 3.209 (95% CI = 3.113–3.305) increase in eGFR. Additionally, with all other factors being constant, a donor-recipient BSA ratio in the 5th percentile compared with 95th percentile (BSA ratio of 0.761 vs. 1.372) was associated with a predicted 1-year eGFR of 50.7 (95% CI = 49.8–51.6) versus 71.2 (95% CI = 70.3–72.1) ml/min per 1.73 m^2^, a difference of 20.5 (95% CI = 19.9–21.1, *P* < 0.001) ml/min per 1.73 m^2^.

Similar associations of worse outcome with smaller donor relative to recipient were observed for 5-year graft survival (aHR for every 0.1 increase in BSA ratio = 0.903, 95% CI = 0.889–0.918, *P* < 0.001; Figure 2e; full model details in Supplementary Table S5 and Supplementary Figure S4), as well as for 1-year graft survival (*P* < 0.001; Supplementary Table S6, splines in Supplementary Figure S5). Adjusting for all other factors, a donor-recipient BSA ratio in the 5th percentile was associated with almost double the hazard for graft failure at 5 years compared with the ratio in the 95th percentile (aHR = 1.93, 95% CI = 1.75–2.13, *P* < 0.001; BSA ratio of 0.761 vs. 1.732). As a sensitivity analysis, the association of donor-recipient BSA ratio with overall graft survival (without censoring for death) at 1 year and 5 years was also assessed. These did not change the overall conclusions (both overall *P* < 0.001; Supplementary Figure S6). Crude 5-year graft survival data stratified by BSA ratio is shown as a Kaplan-Meier plot (Supplementary Figure S3). Smaller donor relative to recipient was also associated with increased risk of delayed graft function incidence (*P* < 0.001; Supplementary Table S7, splines in Supplementary Figure S7).

To further assess the association of donor-recipient BSA ratio with 1-year eGFR, we built an additional model including donor BSA, recipient BSA and donor-recipient BSA ratio simultaneously.^30^ Donor-recipient BSA ratio remained significantly associated with 1-year eGFR (*P* = 0.007). Furthermore, in a multivariable model including interaction terms between donor BSA and recipient BSA, we identified a significant interaction between donor and recipient BSA (interaction *P* = 0.014). These findings provide further evidence that donor-recipient BSA mismatch is associated with outcome beyond the individual associations of donor and recipient BSA. However, interpretation of the individual coefficients from these models is challenging as donor BSA, recipient BSA, and donor-recipient BSA ratio cannot vary freely.

### Interaction Term Analysis

We hypothesized that excess weight gain in adulthood would inflate donor BSA without reflecting increased nephron mass. This was tested using nonlinear interaction analyses, which confirmed a significant interaction between donor-recipient BSA ratio and donor body mass index (BMI; interaction *P* = 0.034; by adding interaction terms to the model in Supplementary Table S4). As seen in Figure 3a, increasing donor-recipient BSA ratio had a greater benefit in donors with low BMI compared with those with high BMI, consistent with our hypothesis. Therefore, this suggests that donor-recipient BSA ratio may not be an optimal factor for kidneys from donors with high BMI.

**Figure 3:**
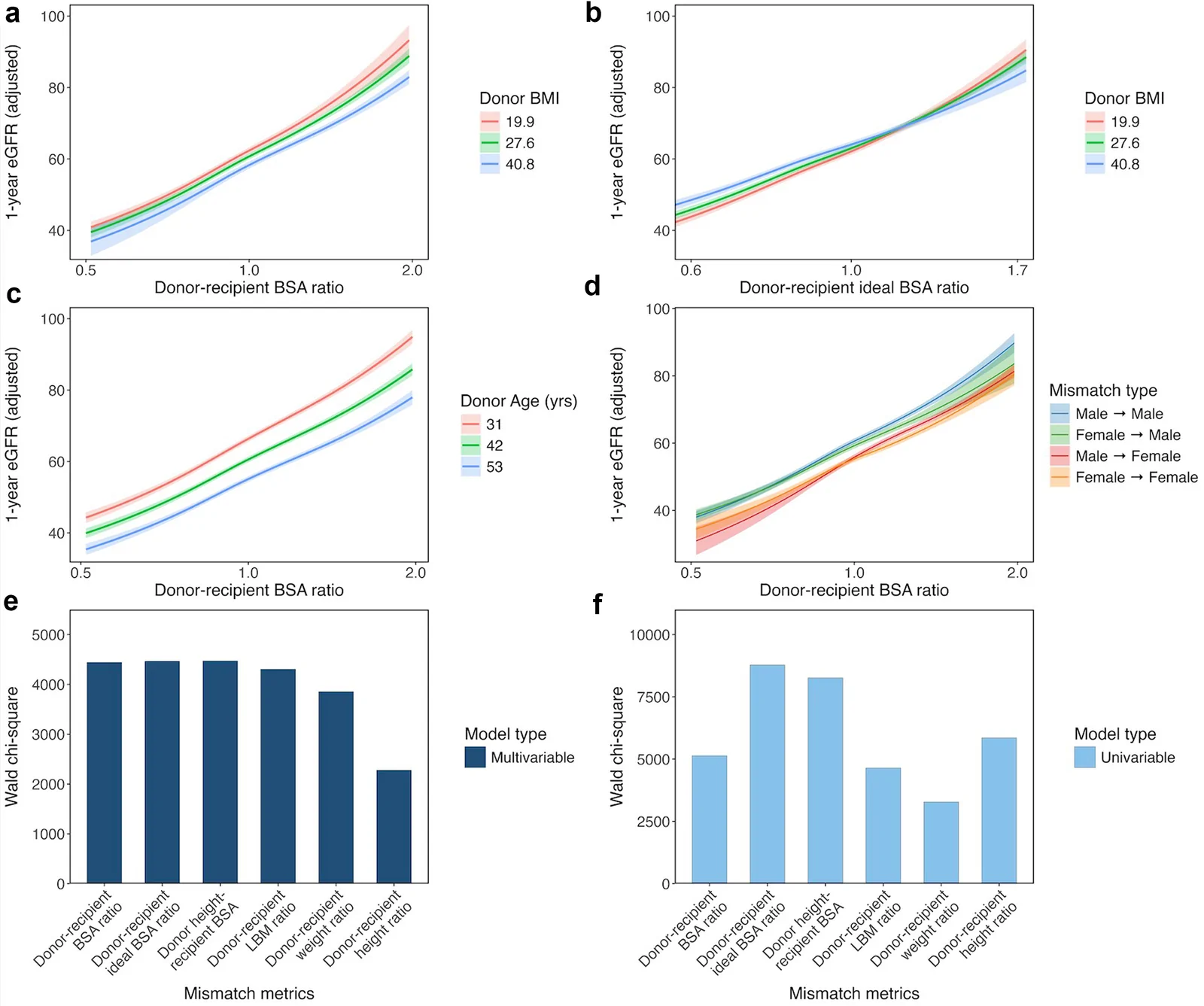
Interaction plots for donor-recipient BSA ratios on 1-year eGFR and Wald chi-square values for various donor-recipient mismatch metrics. (a) interaction between donor-recipient BSA ratio and donor BMI. (b) interaction between ideal donor-recipient BSA ratio and donor BMI. (c) Interaction between donor-recipient BSA ratio and donor age. (d) Interaction between donor-recipient BSA ratio and sex match, all adjusted for covariates in Supplementary Table 4. (e) Wald chi-square values from multivariable models, adjusting for an identical set of covariates. (f) Wald chi-square values for the mismatch metrics from univariable models, A higher Wald chi-square value indicates stronger association with 1-year eGFR. BMI, body mass index; BSA, body surface area; eGFR, estimated glomerular filtration rate; LBM, lean body mass.

To address the issue that donor BSA can overestimate nephron mass in high BMI donors, we calculated the ideal donor BSA (using IBW) and used this value for donors whose actual BSA was greater than ideal BSA. Ideal donor-recipient BSA ratio was also significantly associated with 1-year eGFR (*P* < 0.001, Figure 2d) and 5-year graft survival (*P* < 0.001, Figure 2f). Additionally, the divergence previously seen among high BMI donors with donor-recipient actual BSA ratio is attenuated by ideal donor-recipient BSA ratio (Figure 3b).

We performed interaction analyses between donor-recipient BSA ratio and donor age and recipient age separately. Although the interaction terms were statistically significant, the interaction plots (Figure 3c) showed no clinically significant association. Similarly, within each donor-recipient sex mismatch cohort (male-to-male, female-to-male, female-to-female, and male-to-female), the interaction plots showed no clinically significant differences in the association between donor-recipient BSA ratio and 1-year eGFR across increasing donor ages.

### Accounting for Clustering

Sensitivity analysis was performed to account for variation seen between transplant centers. Repeating the analysis in Supplementary Table S4 using a mixed effects model with random intercepts for transplant center as a random effect remained consistent with our main results. Additionally, we adjusted the model variance for clustering to account for the fact a single donor can donate kidneys to separate recipients; this did not meaningfully change our conclusions.

### Other Measures of Donor-Recipient Size Mismatch

Other measures of donor-recipient size mismatch that we analyzed included donor height-recipient BSA, non-BSA-normalized donor eGFR, and donor-recipient height, weight, and LBM ratios. Donor height-recipient BSA ratio and non-BSA-normalized donor eGFR were both significantly associated with 1-year eGFR and 1-year and 5-year graft survival (all *P* < 0.001, Supplementary Figure S8).

Similar relationships were also seen for donor-recipient height, weight, and LBM ratios, which were significantly associated with 1-year eGFR (height: *P* < 0.001, Supplementary Figure S9A; weight: *P* < 0.01, Supplementary Figure S9D; LBM: *P* < 0.001, Supplementary Figure S9G), 1-year graft survival (height: *P* = 0.021, Supplementary Figure S9B; weight: *P* < 0.001, Supplementary Figure S9E; LBM: *P* < 0.001, Supplementary Figure S9H) and 5-year graft survival (height: *P* < 0.001, Supplementary Figure S9C; weight: *P* < 0.001, Supplementary Figure S9F; LBM: *P* < 0.001, Supplementary Figure S9I).

### Comparing Donor-Recipient Mismatch Metrics

To assess the strength of association of various donor-recipient mismatch metrics with recipient 1-year eGFR outcome, we compared the Wald chi-square value of each metric in both univariable and multivariable models adjusting for an identical set of covariates (comparison of the strength of association between various metrics and outcome are shown in Figure 3e and f). We identified that donor-recipient BSA ratios (actual or ideal) showed the strongest association with outcome compared with other size mismatch metrics (such as height and weight ratios). In adjusted models (including donor BSA and BMI), both actual and ideal donor-recipient BSA ratios were very similarly associated with outcome (Wald chi-square values from multivariable models: actual BSA ratio = 4442.80, ideal BSA ratio = 4464.07). But in univariable models (without donor BSA or BMI), ideal donor-recipient BSA ratio was identified to be more strongly associated with outcome as a stand-alone metric (Wald chi-square values from univariable models: actual BSA ratio = 5136.79, ideal BSA ratio = 8779.80; higher Wald chi-square values indicate stronger association with outcome).

### Importance of Donor-Recipient BSA ratio

To compare the importance of donor-recipient BSA ratio relative to various other variables in the model, we calculated partial F-test and likelihood-ratio chi-square statistics from fully adjusted multivariable models. Figure 4 shows the partial F-test statistics (for 1-year eGFR) and LR chi-square statistics (for 5-year graft survival) for all included factors, demonstrating the relative importance of each variable as a ranked list. Donor-recipient BSA ratio was the second most strongly associated factor with recipient 1-year eGFR, after donor age (Figure 4a; derived from model in Supplementary Table S4). BSA ratio was identified to be more strongly associated than various other variables currently included in the donor risk index or offering schemes in the UK, US, and mainland Europe.^31^, ^32^, ^33^, ^34^, ^35^ Furthermore, donor-recipient BSA ratio was strongly associated with 5-year (Figure 4b; derived from model in Supplementary Table S5) and 1-year graft survival (Supplementary Figure S10; derived from model in Supplementary Table S6). Sensitivity analyses using Wald chi-square statistics remained consistent (Supplementary Figure S11).

**Figure 4:**
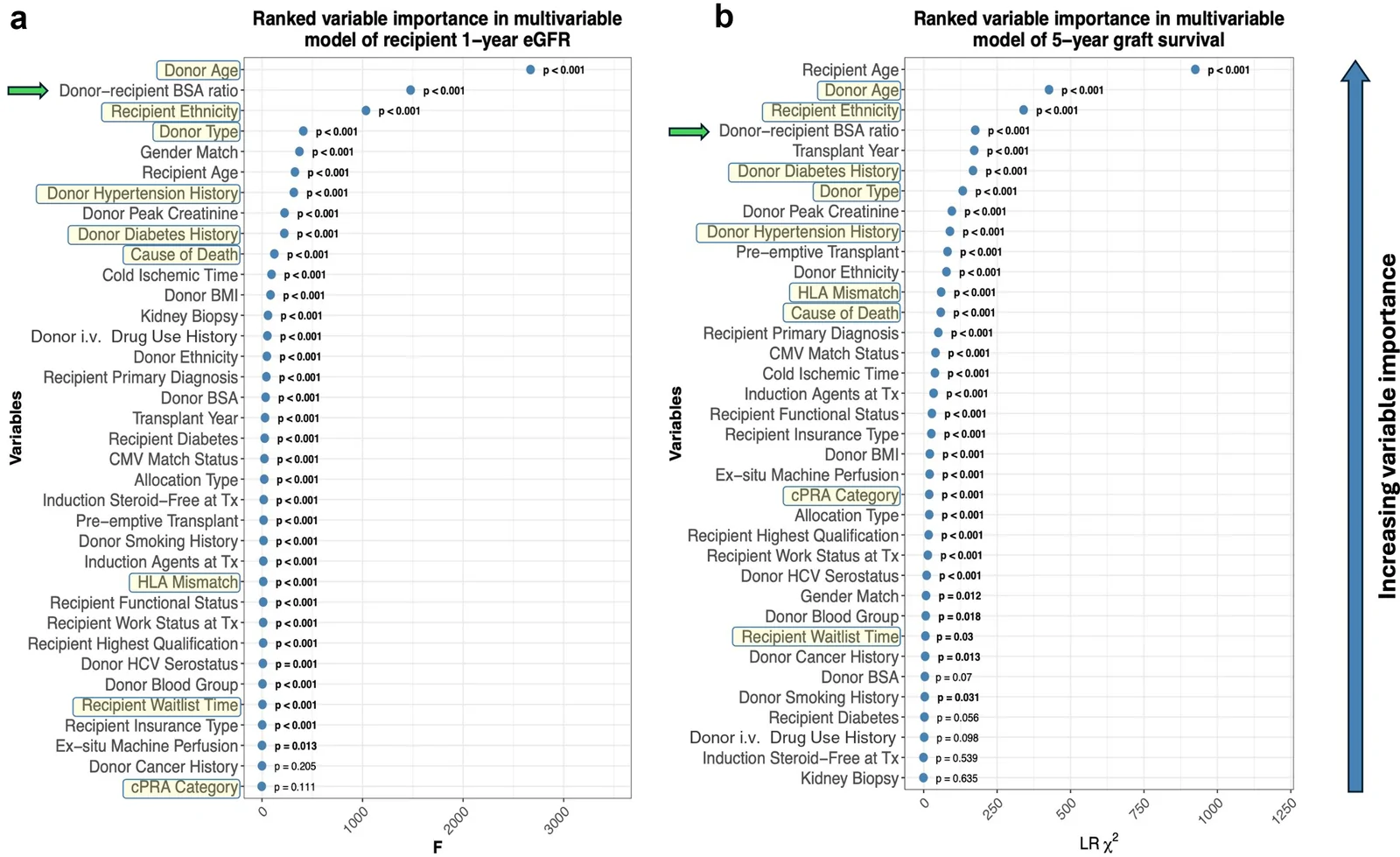
Plot ranking variables from main analysis using ANOVA function from RMS package. Values further right represent greater association of the variable with (a) 1-year eGFR using partial F-tests and (b) 5-year graft survival using LR chi-square values, derived from models in Supplementary Table 4 and Supplementary Table 5 respectively. Factors on the left surrounded by boxes are those currently used in donor-risk indices, offering schemes or/and expanded donor criteria.^31^, ^32^, ^33^, ^34^, ^35^ BSA, body surface area; CMV, cytomegalovirus; HCV, hepatitis C virus; HLA, human leukocyte antigen; Tx, transplantation; cPRA, calculated panel-reactive antibody.

We performed a simulation analysis to estimate how incorporating donor-recipient BSA ratio into kidney offering schemes could help improve equity. As shown in Figure 5, when the offering scheme does not attempt to match donor and recipient size, larger recipients are disadvantaged compared with smaller recipients, with lower estimated 1-year eGFR. Avoiding transplants where ideal donor-recipient BSA mismatch did not exceed a certain threshold resulted in similar predicted 1-year eGFR between smaller and larger recipients (Figure 5), attenuating the disadvantage observed in larger recipients when size mismatch is not considered.

**Figure 5:**
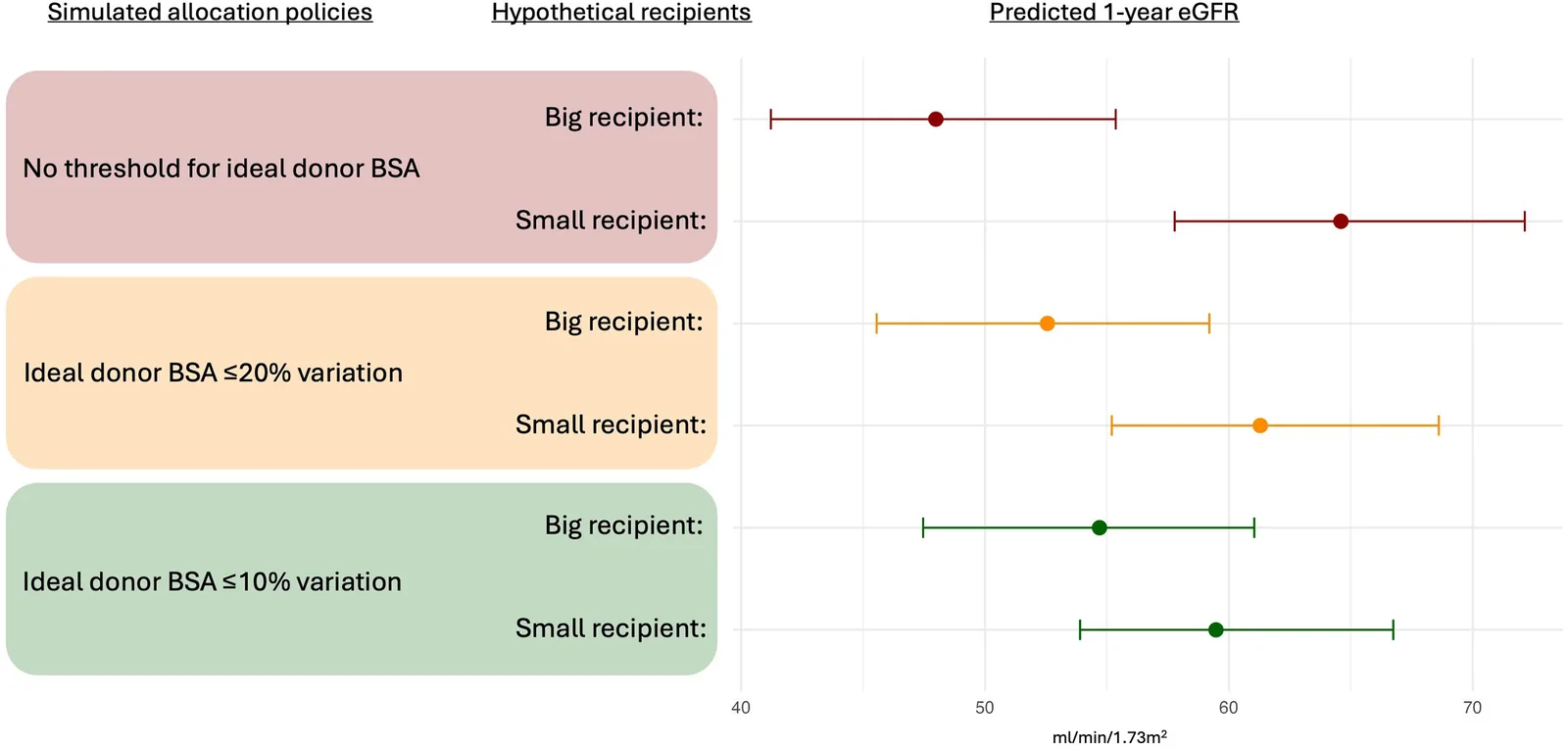
Forest plot showing predicted recipient 1-year eGFR under various simulated offering scheme scenarios. Policies were defined as donor BSA (lowest of ideal or actual donor BSA value) being within 10% or 20% of the recipient’s actual BSA. Outcomes are presented for a small recipient and large recipient, defined by height and weight at the 5th (height 152.4 cm, weight 54.4 kg) and 95th (height 188.0 cm, weight 117.0 kg) percentiles respectively. Dots are median predicted values, with lines showing the interquartile range.

### Dual Transplant Recipient Cohort

We performed additional analyses to see whether the importance of donor-recipient BSA ratio also translated to dual-kidney transplantation. Donor-recipient BSA ratio was significantly associated with 1-year eGFR (*P* < 0.001; Supplementary Figure S12A), 1-year graft survival (*P* = 0.042; Supplementary Figure S12B), and 5-year graft survival (*P* = 0.003; Supplementary Figure S12C). Similar trends of consistently worse outcome were observed with small donor kidneys in larger recipients.

### Creation of Web Application

Following the analyses above, we developed a freely available web application using the shinyapp framework to calculate (ideal) donor-recipient BSA ratios,^36^^,^^37^ available at https://dhareshraj.shinyapps.io/Kidney_donor-recipient_size_matching_calculator/. The application allows users to input donor and recipient anthropometric variables (height and weight) to calculate individual BSAs and donor-recipient BSA ratios.

## Discussion

To our knowledge, this is the largest study to date analyzing the importance of donor-recipient size mismatch in kidney transplant outcomes, and the first to employ nonlinear modelling. We demonstrated that donor-recipient BSA ratio is a critical factor associated with post-transplant outcome, with kidneys from smaller donors into larger recipients consistently showing worse outcome. Nonlinear interaction analyses revealed that the clinical association of BSA ratio remained consistent regardless of donor age or donor-recipient sex mismatch. This suggests that donor-recipient size mismatch is a crucial but often overlooked factor; it should be incorporated into offering schemes to optimize kidney offering and promote equity in recipient outcomes, and be included as a potential critical covariate or confounder in both prospective clinical trials and observational studies.

In this study, we wanted the donor-recipient size mismatch metric to represent the mismatch between available donor nephron mass (the numerator) and required recipient nephron mass (the denominator). Our primary mismatch metric of interest was donor-recipient BSA ratio, as BSA is a well-established anthropomorphic surrogate measure for functional nephron mass.^4^

We identified that donor-recipient BSA ratio was 1 of the most strongly associated factors with outcome (Figure 4). It was the second most strongly associated factor with recipient 1-year eGFR, only after donor age, out of 36 examined factors. It was much more strongly associated with outcome than several other variables currently being used in offering schemes and risk indices in the US, UK, and Europe, such as donor cause of death, donor hypertension, donor diabetes, donor i.v. drug user, human leukocyte antigen mismatch, calculated panel reactive antibody score, recipient waitlist time, recipient age, and recipient dialysis status.

Multivariable models analyzing donor-recipient BSA ratio were also adjusted for donor BSA. This approach was used to identify the independent association of size mismatch, separate from the association of the donor factor itself. For example, kidneys from donors with greater nephron mass (large BSA) may lead to better outcomes in general, regardless of the recipient’s nephron requirement (recipient BSA). The fact that the degree of size mismatch was a critical variable, after adjusting for donor size, emphasizes the association of donor-recipient matching with ramifications for kidney offering schemes.

We hypothesized that the biological mechanism for our findings may be explained by the nephron dosing hypothesis.^3^ Kidneys from smaller donors may contain fewer functioning nephrons and therefore provide a lower nephron mass relative to the metabolic demands of larger recipients. This mismatch may lead to hyperfiltration-mediated injury and reduced graft function. However, further research is required to confirm the precise mechanisms underlying the observed clinical associations in this study.

We compared the Wald chi-square values of various donor-recipient size mismatch metrics to assess how strongly they are associated with post-transplant outcomes. In univariable analyses, ideal donor-recipient BSA ratio was more strongly associated with outcome compared with actual donor-recipient BSA ratio. However, in multivariable analyses adjusting for donor BSA and BMI, both actual and ideal BSA ratios became very similar. Therefore, if a stand-alone factor is needed (for example in kidney offering), then ideal donor-recipient BSA shows the strongest association with outcome. In multivariable models adjusting for donor BMI and BSA (for example in prospective clinical trials and observational studies), either actual or ideal BSA ratio can be used.

Existing research on the association of donor-recipient size mismatch with outcome have been mixed and inconclusive.^5^^,^^6^^,^^38^^,^^39^ A recent independent review that included studies from the past 5 decades concluded that there is a “critical gap in the transplant literature—despite decades of research and biological plausibility rooted in the nephron dosing hypothesis, there is no consensus on how to define or operationalize size mismatch”.^5^ All included studies in this review were reported to have high or very high risk of bias, indicating a need for high quality evidence in this area of research. Although the review reported the use of various size mismatch metrics, there is still no consensus regarding the most optimal metric.

Lepeytre *et al.*^6^ also analyzed the association of donor-recipient BSA ratio using the UNOS cohort (2000–2018). Although it demonstrated that BSA ratio was an important factor associated with kidney transplant outcomes, there are some limitations associated with this study. They modelled donor-recipient BSA ratio as a categorical variable with arbitrary groups (< 0.8, 0.8–0.89, 0.9–0.99, ≥ 1.0), and did not analyze whether increasing BSA ratio (large donor with respect to recipient) lead to a continuous improvement in outcomes. It only analyzed BSA ratio as a measure of donor-recipient size mismatch.

Additionally, Lepeytre *et al.*^6^ reported a statistically significant 3-way interaction between donor-recipient BSA ratio, donor age, and recipient age, suggesting that the adverse association of severe size mismatch (donor-recipient BSA ratio < 0.8) may be more pronounced with increasing donor and recipient age. In our study, we identified statistically significant interaction (*P* < 0.05) between BSA ratio and both donor and recipient age, although deemed these findings to be not clinically significant (Figure 3c). This is consistent with the relatively small differences and overlapping CIs reported by Lepeytre *et al.*^6^ Furthermore, Lepeytre *et al.*^6^ included donors aged ≥ 5, thereby allowing more extreme donor-recipient BSA mismatches than our adult donor cohort (aged ≥ 18 years).

In this study, we have demonstrated that donor-recipient BSA ratio is strongly associated with outcome. Unlike previous studies, we employed nonlinear modelling using restricted cubic splines to analyze the size mismatch metrics. This approach has been identified as a superior modelling approach to preserve the true nature of the association, rather than assuming a linear relationship or splitting a continuous factor into arbitrary groups.^28^ We analyzed multiple size mismatch metrics and compared their strengths of association with outcome. To our knowledge, we are the first to perform a simulation analysis to show the importance of donor-recipient size mismatch in improving equity.

One of the limitations of this study is that donor and recipient BSA were used as surrogates for nephron mass and nephron demand, respectively. However, neither are perfect measures, and other factors such as muscle mass may play an important role in determining donor available nephron mass and recipient nephron mass requirement. Furthermore, BSA was calculated from height and weight using the Du Bois formula, which provides an estimate rather than a precise measurement. However, this reflects real-world practice, where directly measuring the true BSA or nephron mass is not feasible.

Future work is needed to explore more accurate methods of estimating nephron mass, both before retrieval and ex situ. For instance, donor cross-sectional imaging could be used to measure kidney volume and better estimate nephron mass before retrieval, potentially influencing offering decisions. Additionally, ex-situ photographic analysis of kidneys to measure their size or surface area, alongside functional assessment, could provide a more accurate estimate of viable nephron mass.

Another limitation is the retrospective design of the study and potential for selection bias. However, we adjusted for a wide range of covariates, which was possible because of the large cohort size. There is also an inevitable degree of missing data, but robust techniques were used to impute this data. Finally, we performed exploratory analyses on how placing a greater emphasis on donor-recipient size matching in offering schemes would affect post-transplant outcomes and equity. However, more formal simulation studies are needed to explore this.

## Conclusion

We have shown that donor-recipient size mismatch is strongly associated with post-transplant outcomes, but is overlooked in the majority of current offering schemes and risk indices. Donor-recipient BSA ratio was strongly associated with recipient 1-year eGFR, as well as with 1- and 5-year graft survival. For 1-year eGFR, it was the second most strongly associated factor with outcome only after donor age, and more important than most other variables currently included in offering schemes and/or risk indices. When analyzed as a single stand-alone factor, ideal donor-recipient BSA showed the strongest association with outcome among all mismatch metrics examined. These findings support incorporating donor-recipient BSA ratio into offering schemes to optimize kidney offering, and including it as a potential critical covariate or confounder in both prospective clinical trials and observational studies.

## Disclosure

All the authors declared no competing interests.

## Supplementary Material

Supplementary file (PDF) **Figure S1.** Missing data pattern. **Figure S2.** Association of factors with recipient 1-year eGFR incidence using restricted cubic splines with 4 knots. **Figure S3.** Kaplan-Meier curve of 5-year graft survival stratified by donor-recipient BSA ratio. **Figure S4.** Association of factors with recipient 5-year graft survival using restricted cubic splines with 4 knots**. Figure S5**. Association of factors with recipient 1-year graft survival using restricted cubic splines with 4 knots. **Figure S6.** Restricted cubic splines showing the association between donor-recipient and (A) 1-year overall graft survival (B) and 5-year overall graft survival (without censoring for death). **Figure S7.** Association of factors with delayed graft function using restricted cubic splines with 4 knots. **Figure S8.** Association of donor height-recipient BSA mismatch. **Figure S9.** Association of donor-recipient height ratio. **Figure S10.** Plot showing LR chi-square statistics for variables from main analysis using ANOVA function from RMS package. **Figure S11.** Plot Wald chi-square ranking variables from main analysis using ANOVA function from RMS package. **Figure S12.** Association of donor-recipient BSA ratio in dual kidney transplants. **Table S1.** Complete list of covariates included in all multivariable analyses. **Table S2.** Full extended cohort demographics. **Table S3.** Full definitions of recipient functional status groups. **Table S4.** Multivariable linear model including donor-recipient BSA ratio for 1-year. **Table S5.** Multivariable cox model including donor-recipient BSA ratio for 5-years. **Table S6.** Multivariable cox model including donor-recipient BSA ratio for 1-year. **Table S7 (Excel).** Multivariable cox model including donor-recipient BSA ratio for delayed graft function incidence. STROBE checklist.

Table S7 (Excel)
