# Supplementary material for: Donor-Recipient Size Mismatch is a Critical Determinant of Kidney Transplant Outcome
Source: Kidney Int Rep. 2026 Sep 23;11(12):107100. doi: 10.1016/j.ekir.2026.107100 (PMC13697278; doi:10.1016/j.ekir.2026.107100)
Supplement: Supplementary file 1 [file mmc1.pdf]

Supplementary figures

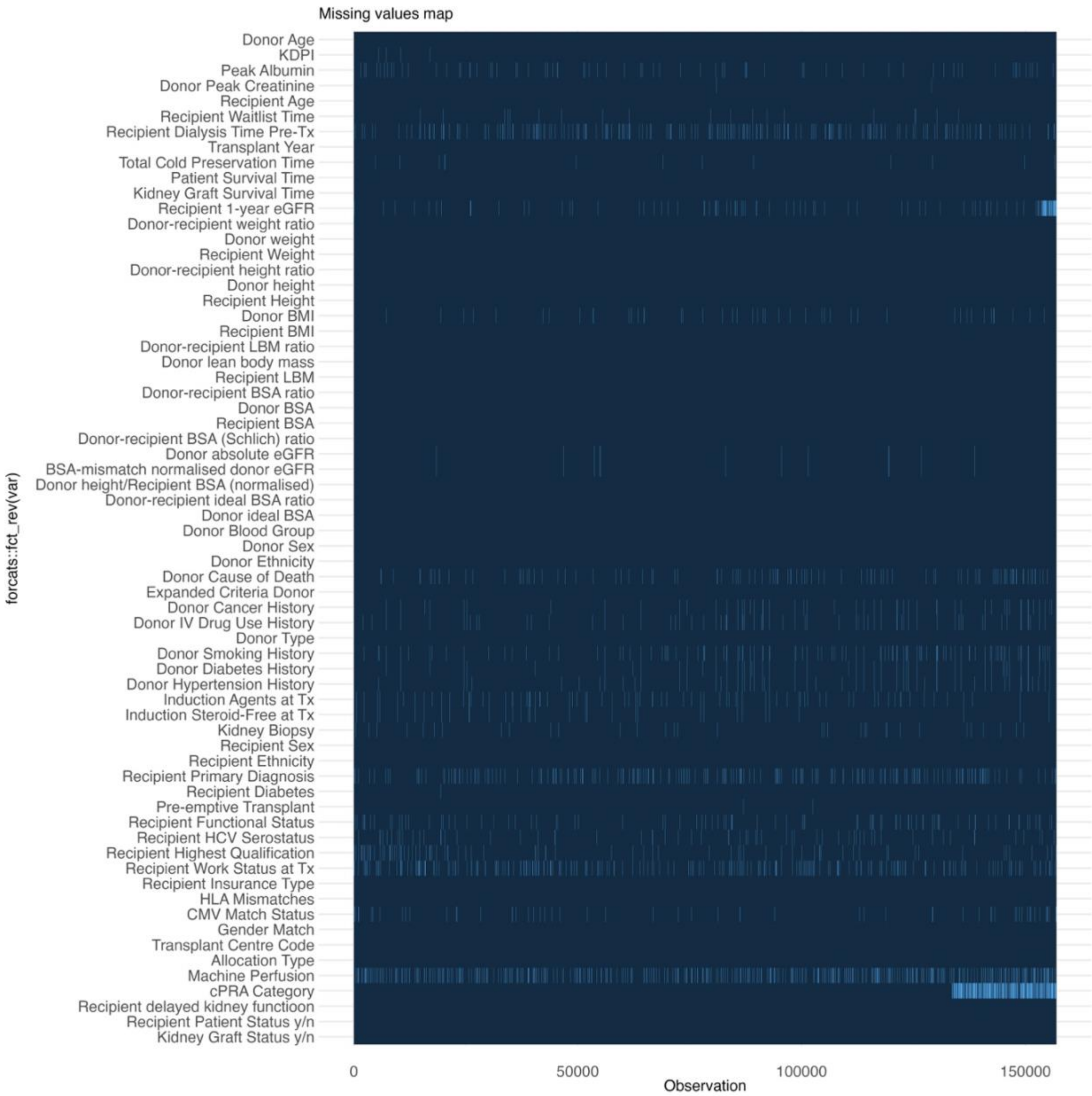

Supplementary Figure 1: Missing data pattern. The x-axis represents the pattern in missing data and the y-axis represents various variables. Missing data is shown in light blue and available data in dark blue.

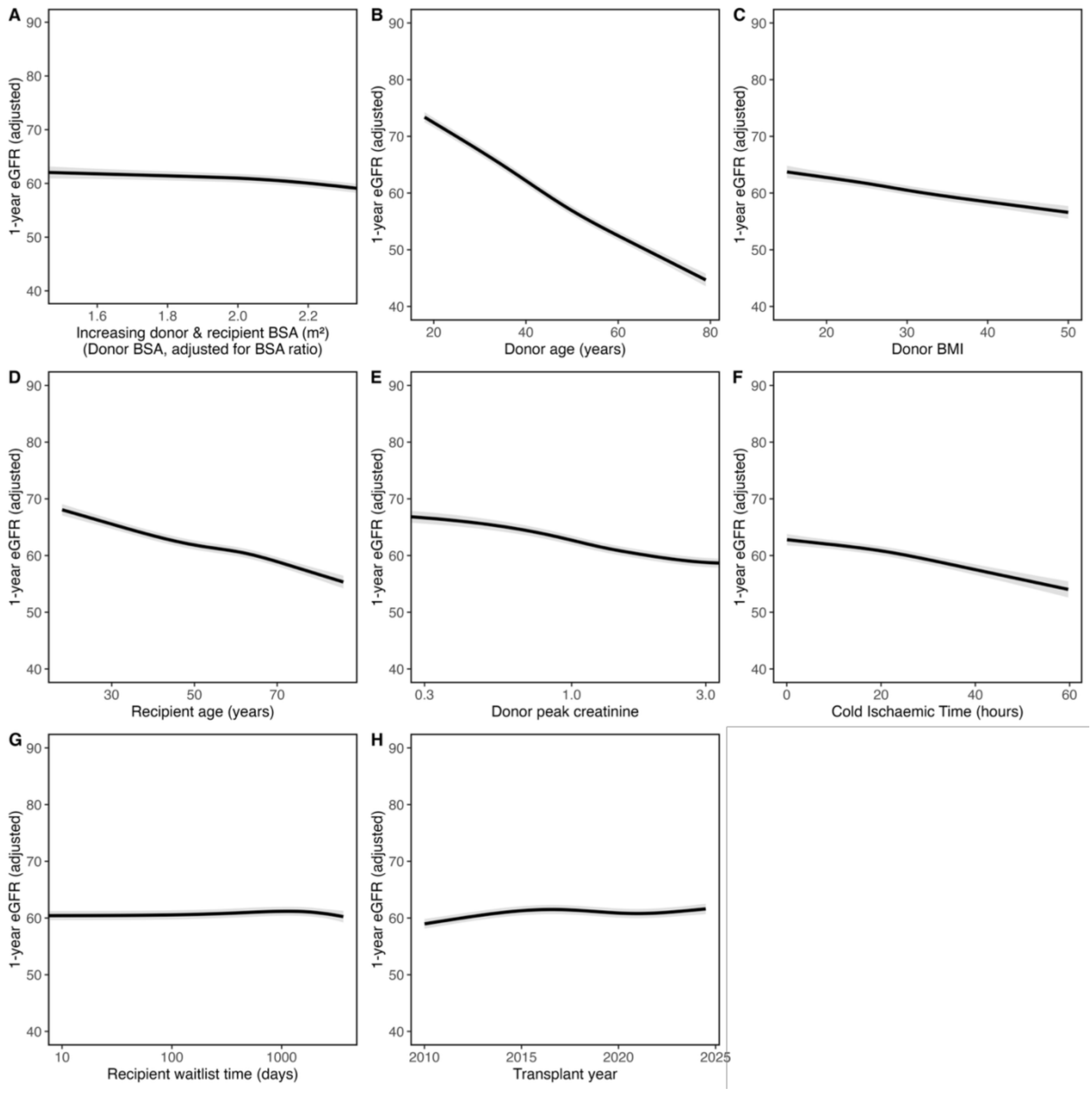

Supplementary Figure 2: Association of factors with recipient 1-year eGFR incidence using restricted cubic splines with 4 knots. Grey lines represent the adjusted change in outcome, with grey shaded areas representing the 95% confidence intervals. Associations between (A) increasing donor & recipient BSA, (B) donor age, (C) donor BMI, (D) recipient age, (E) donor peak creatinine, (F) cold ischemic time, (G) recipient waitlist time and (H) transplant year on 1-year eGFR (derived from model in ***Error! Reference source not found.****).*

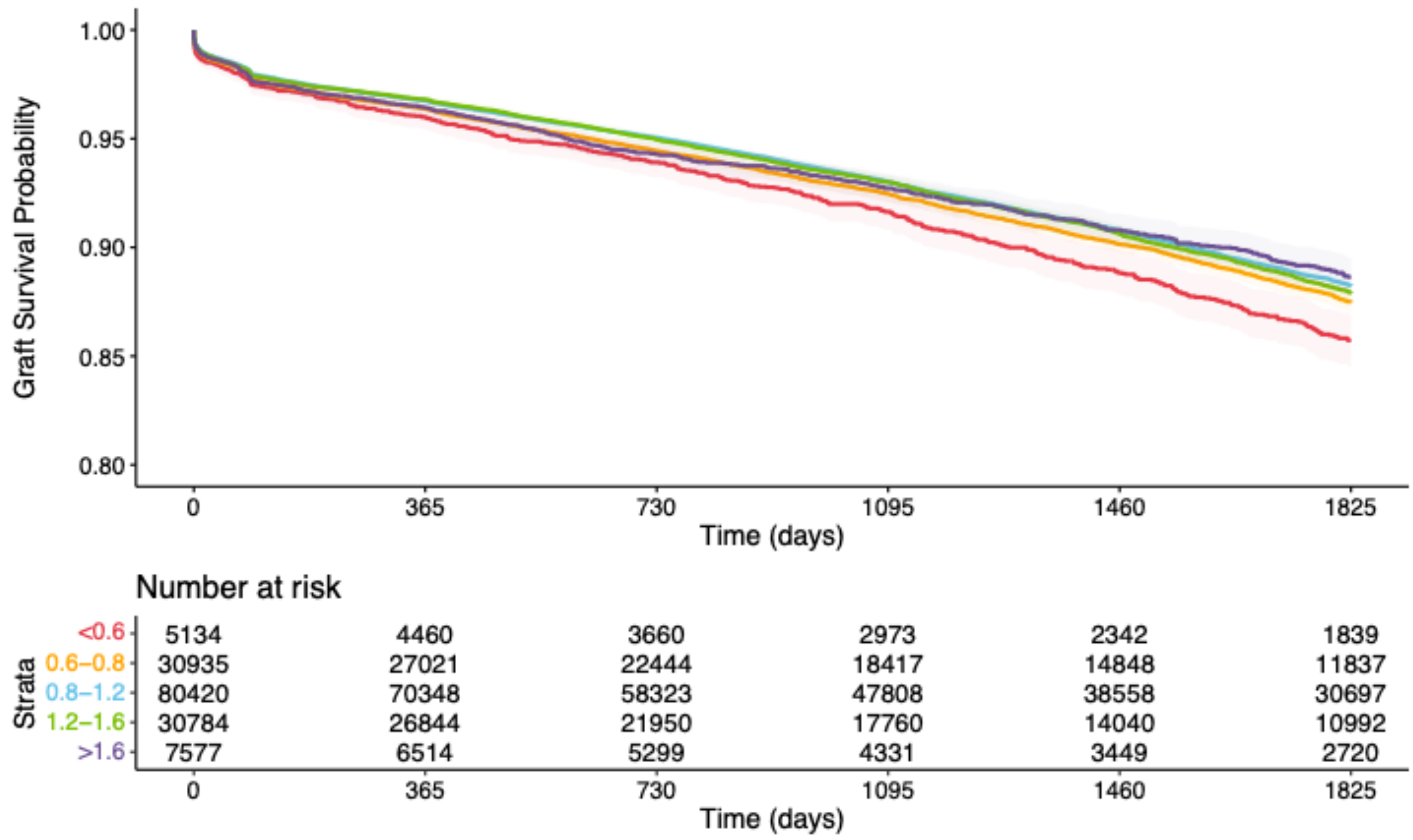

Supplementary Figure 3: Above – Kaplan-Meier curve of 5-year graft survival stratified by donor-recipient BSA ratio. Below – Number at risk of graft failure over the 5-year period.

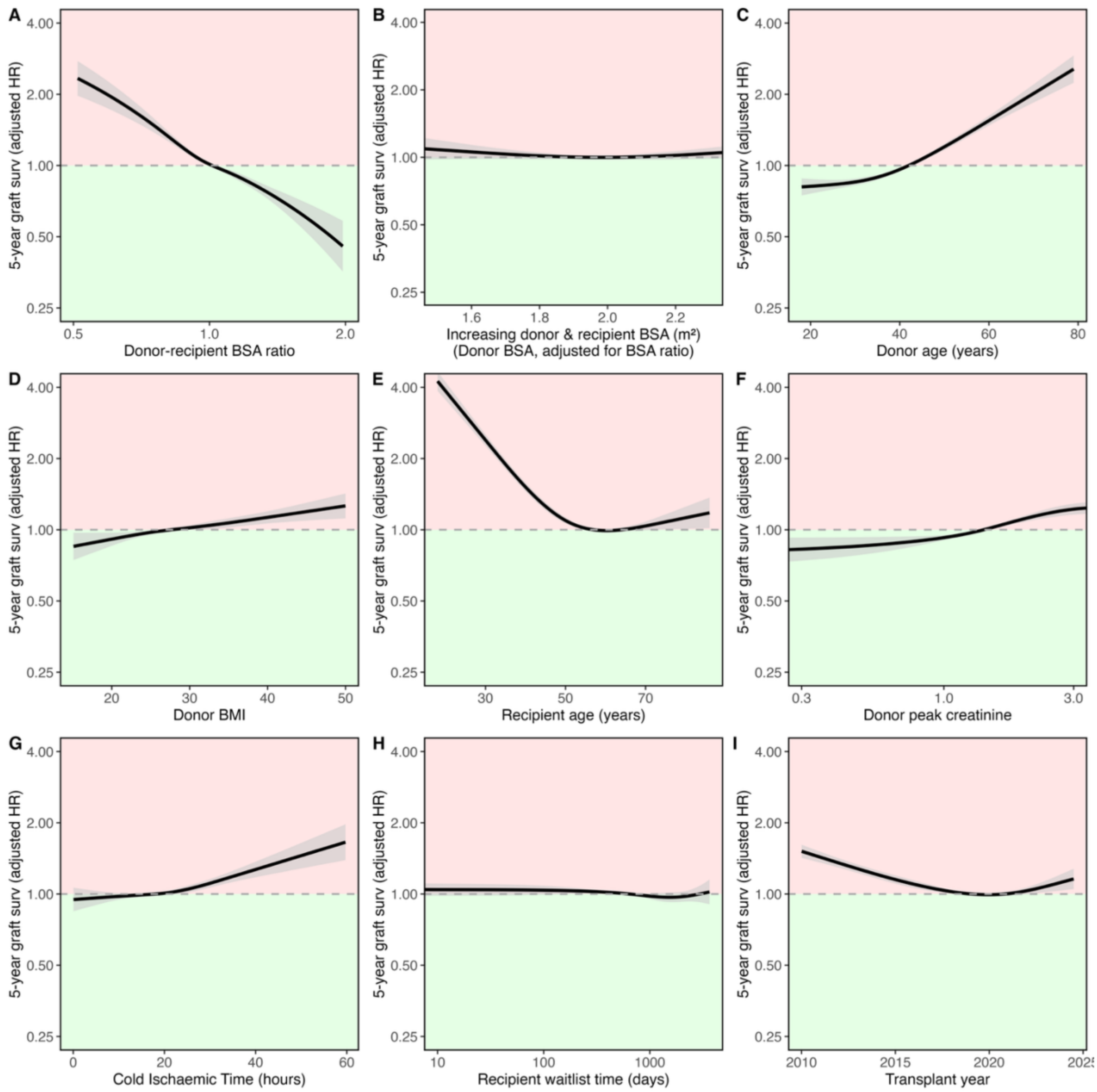

Supplementary Figure 4: Association of factors with recipient 5-year graft survival using restricted cubic splines with 4 knots. Grey lines represent the adjusted change in outcome, with grey shaded areas representing the 95% confidence intervals. The y axis Is log scaled. Associations between (A) donor-recipient BSA ratio, (B) increasing donor & recipient BSA, (C) donor age, (D) donor BMI, (E) recipient age, (F) donor peak creatinine, (G) cold ischemic time, (H) recipient waitlist time and (I) transplant year on 5-year graft survival (derived from model in ***Error! Reference source not found.****). The green region represents superior outcome compared to the reference, while the red region signifies inferior outcome.*

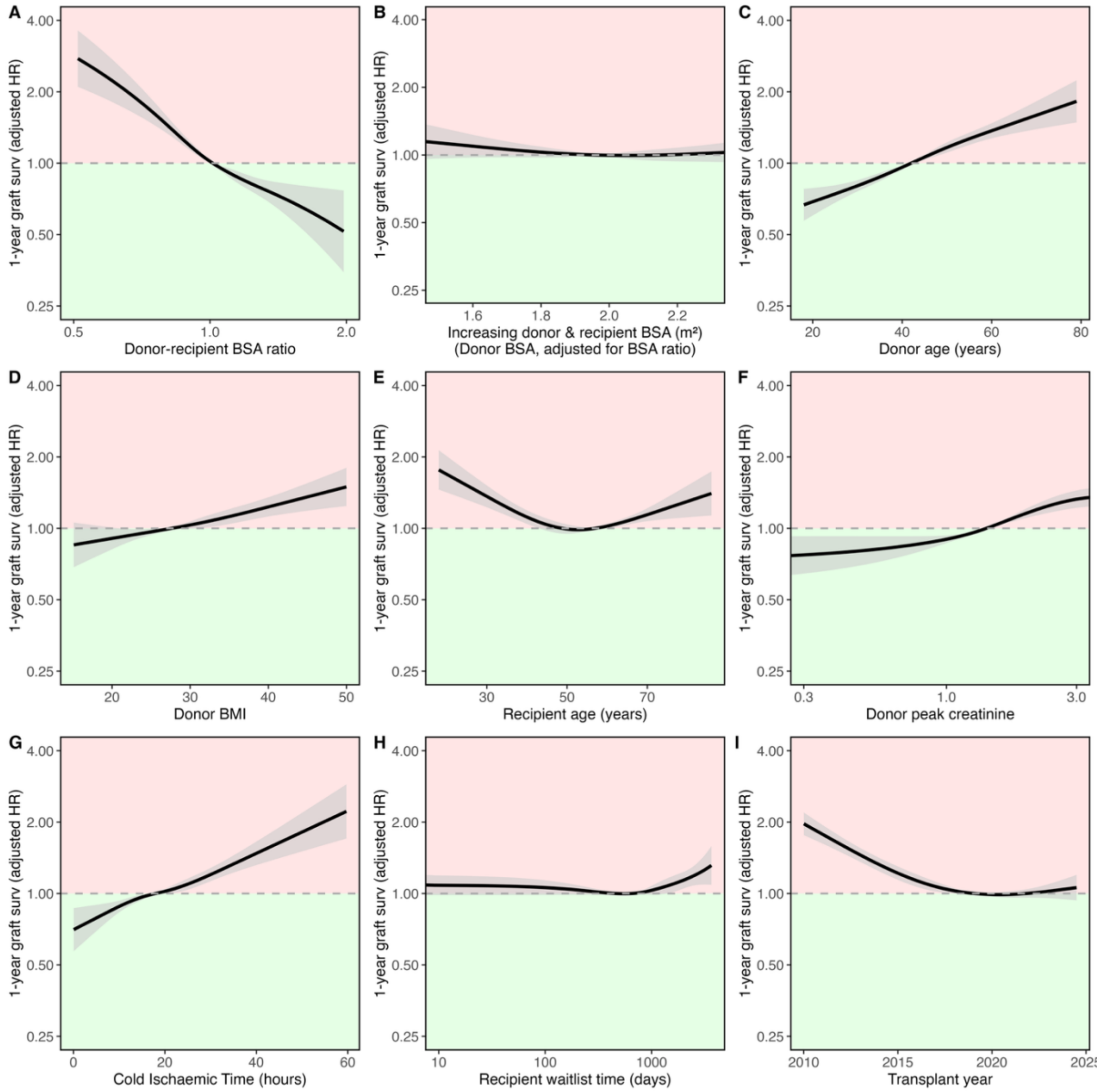

Supplementary Figure 5: Association of factors with recipient 1-year graft survival using restricted cubic splines with 4 knots. Grey lines represent the adjusted change in outcome, with grey shaded areas representing the 95% confidence intervals. The y axis is log scaled. Associations between (A) donor-recipient BSA ratio, (B) increasing donor & recipient BSA, (C) donor age, (D) donor BMI, (E) recipient age, (F) donor peak creatinine, (G) cold ischemic time, (H) recipient waitlist time and (I) transplant year on 1-year graft survival (derived from model in **Error! Reference source not found.**. The green region represents superior outcome compared to the reference, while the red region signifies inferior outcome.

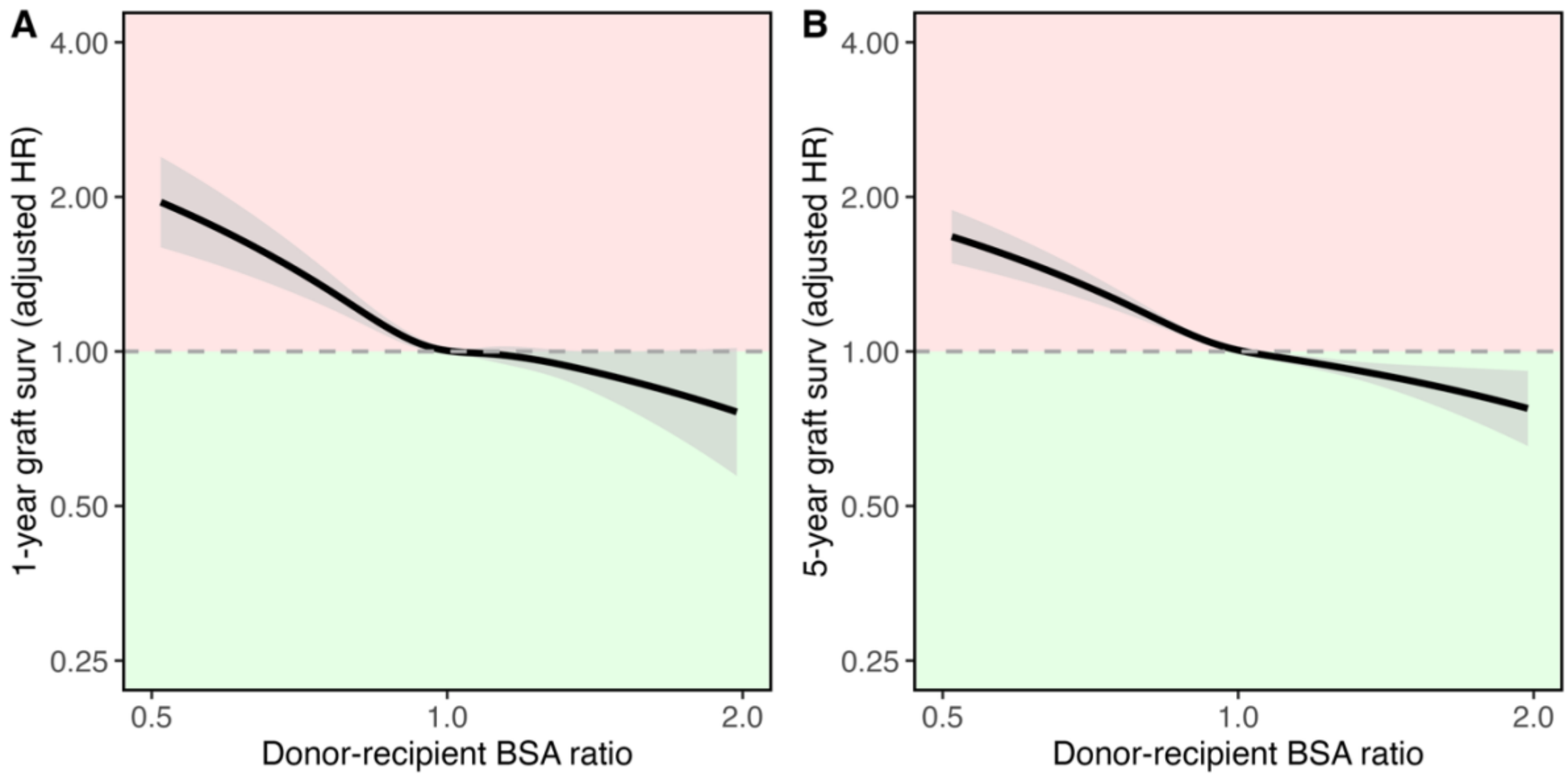

Supplementary Figure 6: Restricted cubic splines showing the association between donor-recipient and (A) 1-year overall graft survival (B) and 5-year overall graft survival (without censoring for death).

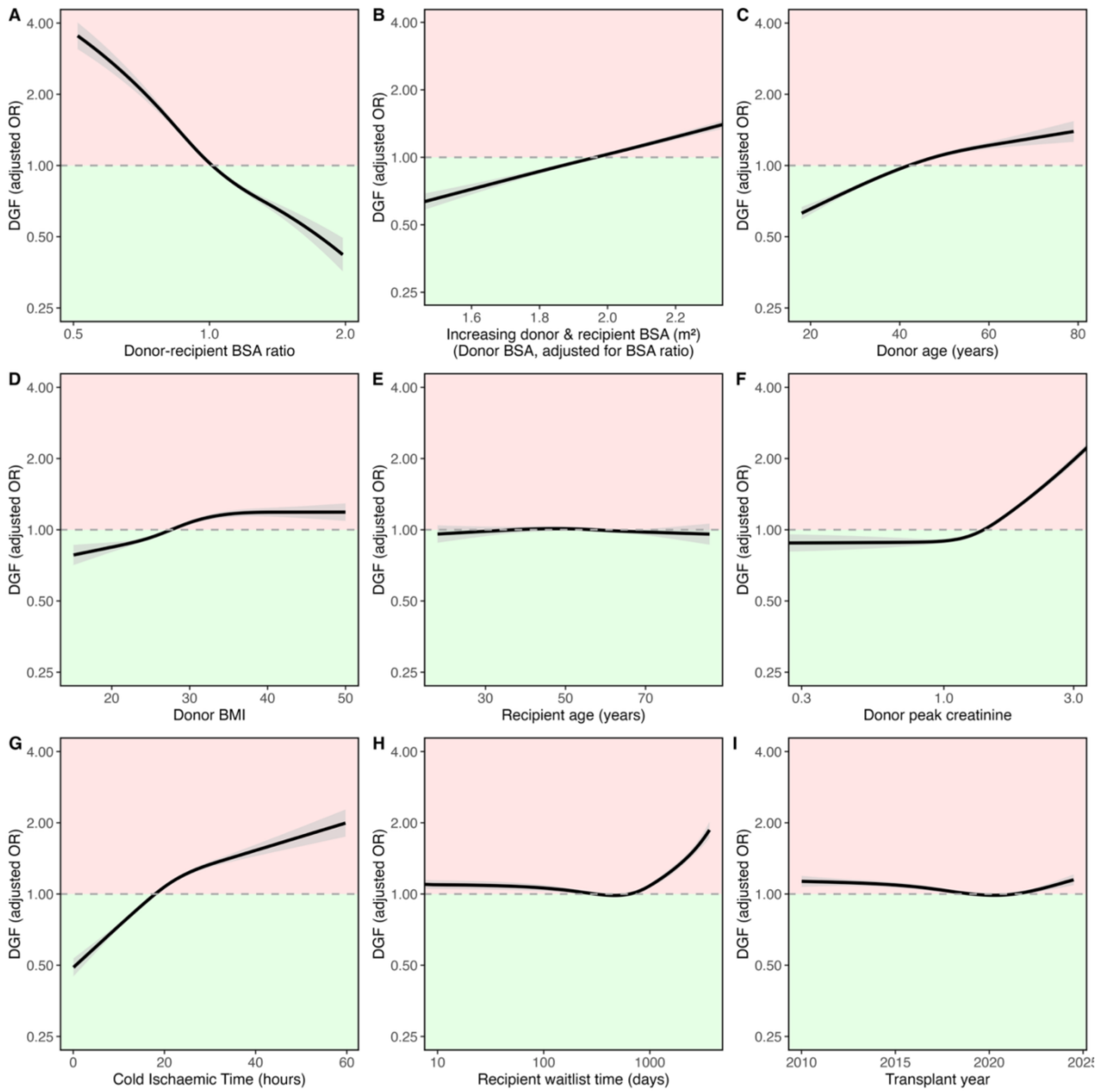

Supplementary Figure 7: Association of factors with delayed graft function using restricted cubic splines with 4 knots. Grey lines represent the adjusted change in outcome, with grey shaded areas representing the 95% confidence intervals. The y axis Is log scaled. Associations between (A) donor-recipient BSA ratio, (B) increasing donor & recipient BSA, (C) donor age, (D) donor BMI, (E) recipient age, (F) donor peak creatinine, (G) cold ischemic time, (H) recipient waitlist time and (I) transplant year on delayed graft function (derived from model in **Error! Reference source not found.**). The green region represents superior outcome compared to the reference, while the red region signifies inferior outcome.

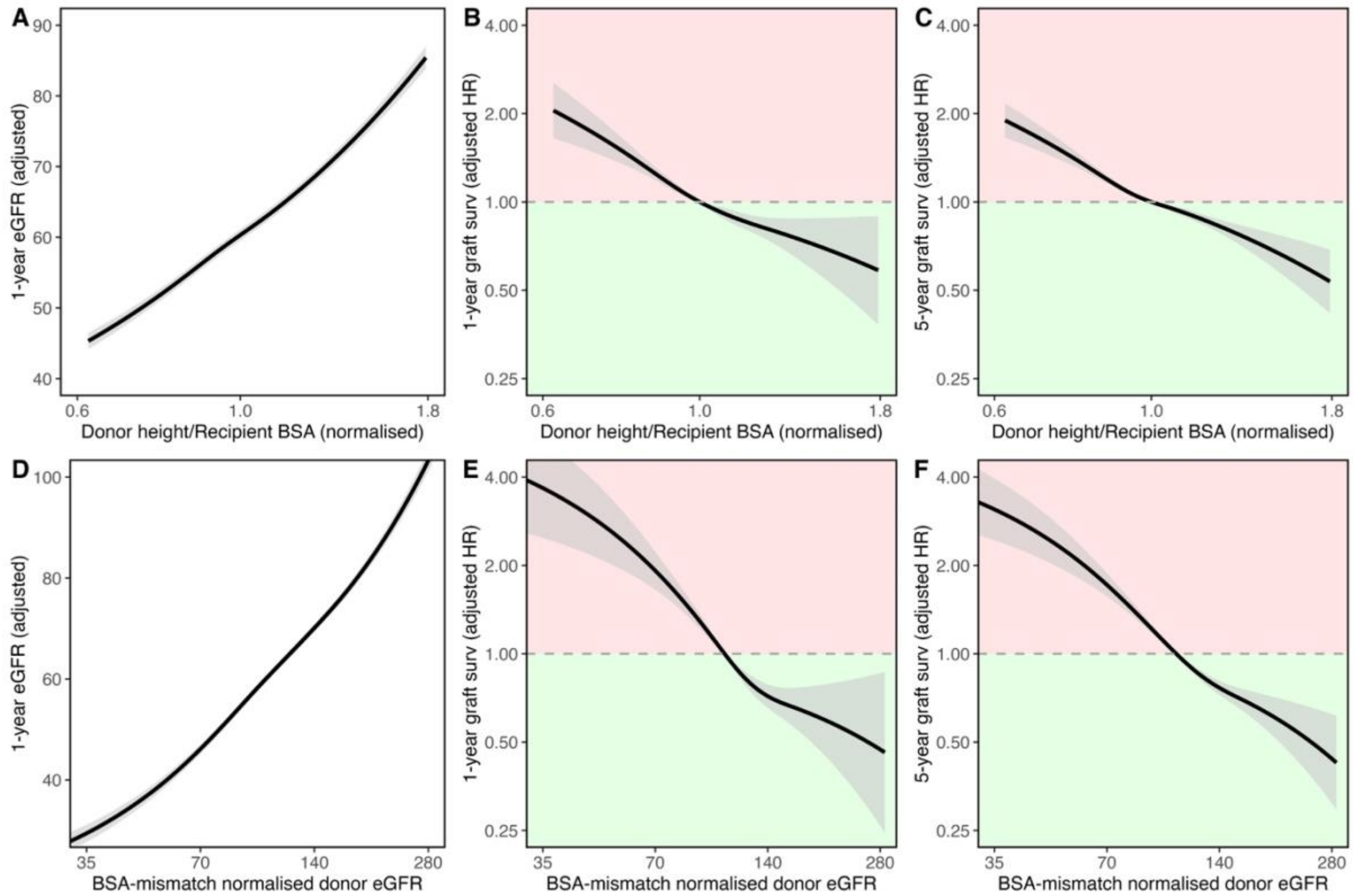

Supplementary Figure 8: Association of donor height-recipient BSA mismatch on (A) 1-year eGFR, (B) 1-year graft survival and (C) 5-year graft survival. Association of non-BSA-normalised donor eGFR on (D) 1-year recipient eGFR, (E) 1-year graft survival and (F) 5-year graft survival. Grey lines represent the adjusted change in outcome, with grey shaded areas representing the 95% confidence intervals. The green region represents superior outcome compared to the reference, while the red region signifies inferior outcome.

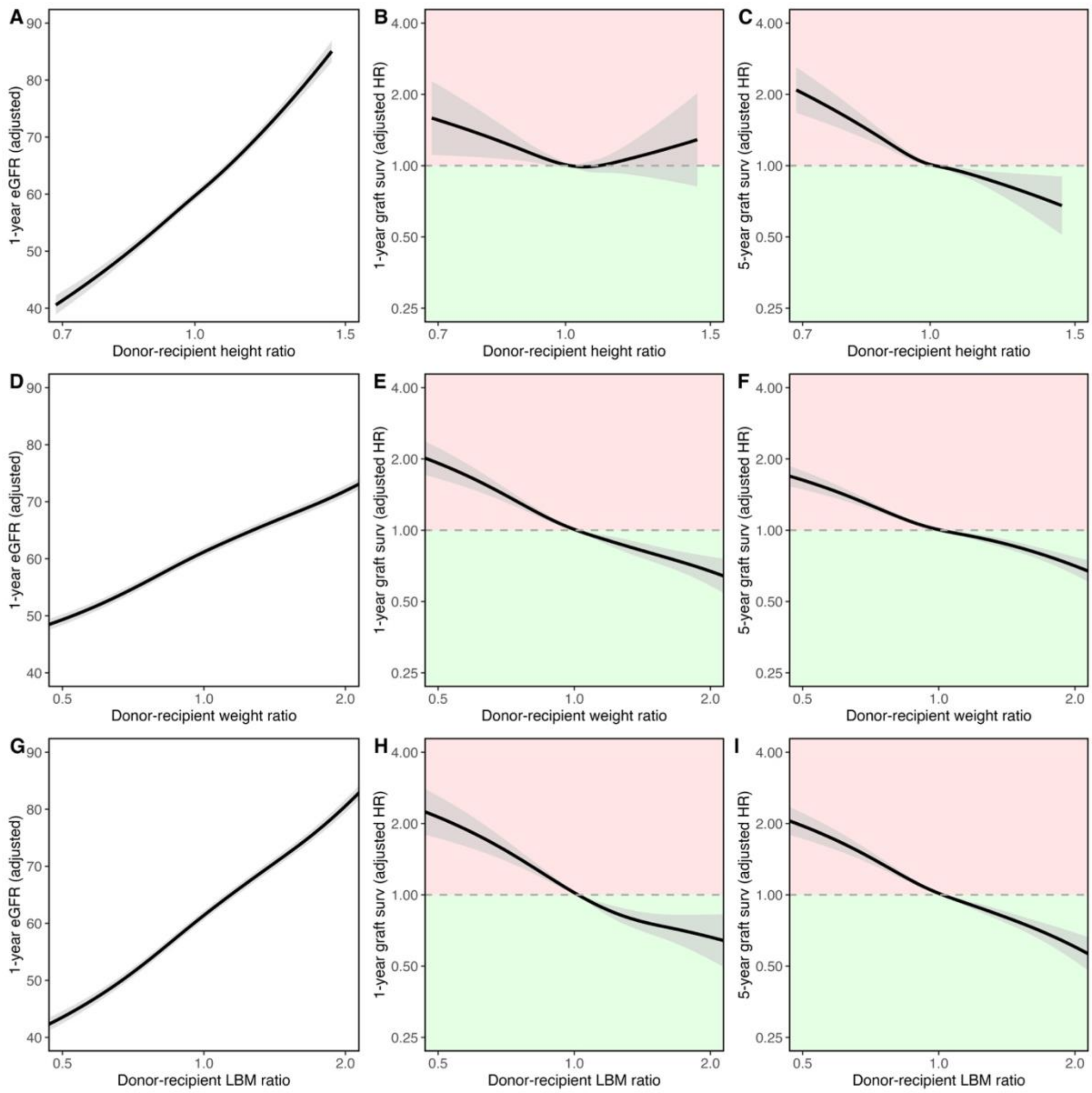

Supplementary Figure 9: Association of donor-recipient height ratio on (A) 1-year eGFR, (B) 1-year graft survival and (C) 5-year graft survival. Association of donor-recipient weight ratio on (D) 1-year recipient eGFR, (E) 1-year graft survival and (F) 5-year graft survival. Association of donor-recipient lean body mass ratio on (G) 1-year recipient eGFR, (H) 1-year graft survival and (I) 5-year graft survival. Grey lines represent the adjusted change in outcome, with grey shaded areas representing the 95% confidence intervals. The green region represents superior outcome compared to the reference, while the red region signifies inferior outcome.

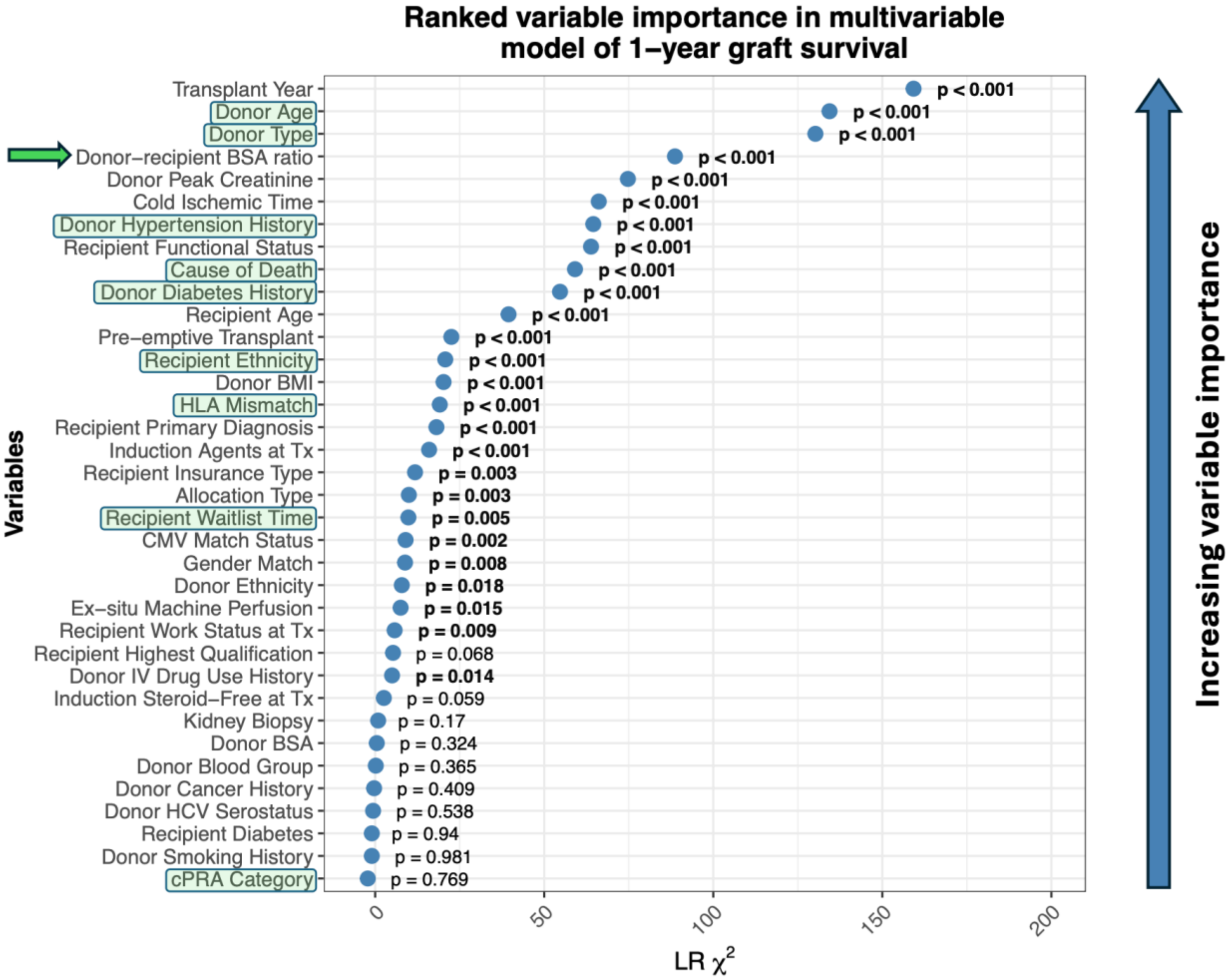

Supplementary Figure 10: Plot showing LR chi-square statistics for variables from main analysis using ANOVA function from RMS package. Values further right represent greater strength of the association of the variable with 1-year graft survival, derived from model in ***Error! Reference source not found.****. Factors highlighted are those currently used in donor-risk index, offering schemes or/and expanded donor criteria (26–30).*

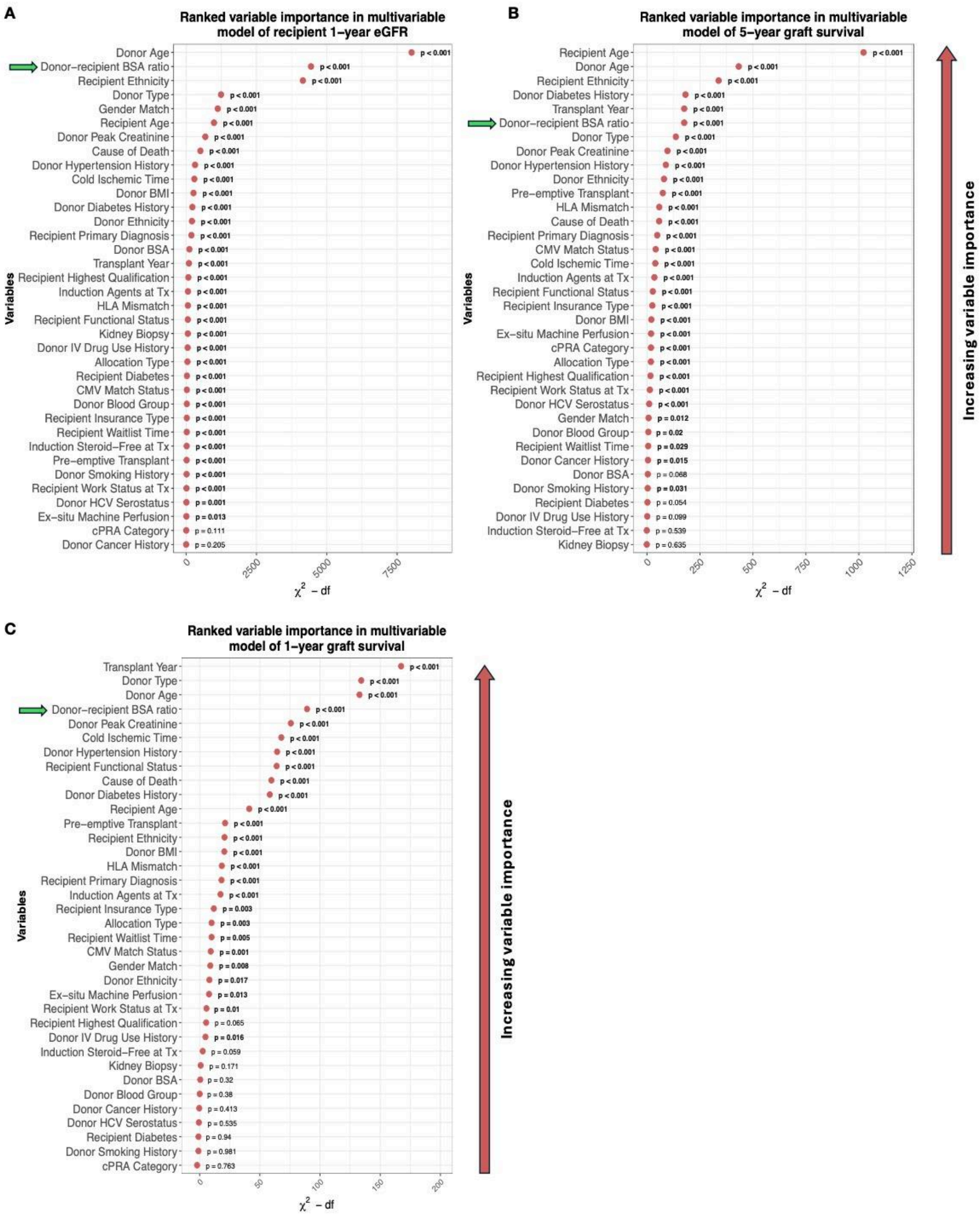

Supplementary Figure 11: Plot Wald chi-squrare ranking variables from main analysis using ANOVA function from RMS package. Values further right represent greater strength of association of the variable in predicting (A) 1-year eGFR, (B) 5-year graft survival and (C) 1-year graft survival.

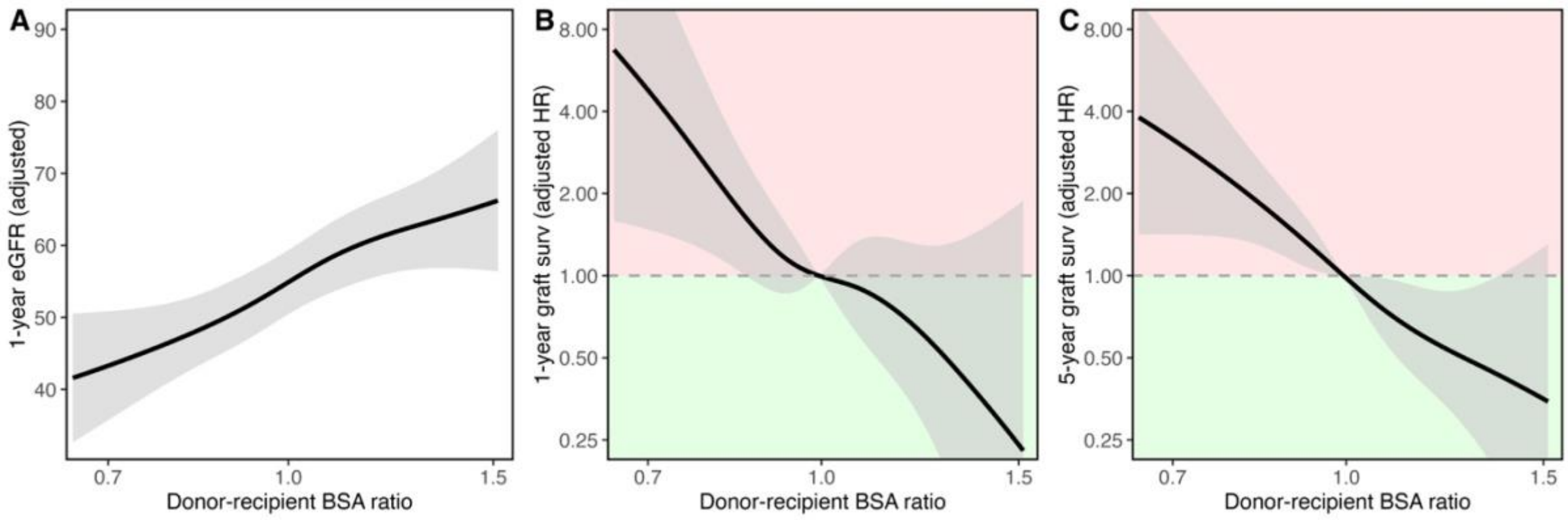

Supplementary Figure 12: Association of donor-recipient BSA ratio in dual kidney transplants on (A) 1-year eGFR, (B) 1-year graft survival and (C) 5-year graft survival. Grey lines represent the adjusted change in outcome, with grey shaded areas representing the 95% confidence intervals. The green region represents superior outcome compared to the reference, while the red region signifies inferior outcome.

STROBE Statement—checklist of items that should be included in reports of observational studies

	Item No.	Recommendation	Page No.	Relevant text from manuscript
**Title and abstract**	1	(*a*) Indicate the study's design with a commonly used term in the title or the abstract	2	*"Methods: Population cohort study using UNOS data (2010-2024…"*
		(*b*) Provide in the abstract an informative and balanced summary of what was done and what was found	2	*"Population cohort study using UNOS data… donor-recipient BSA-ratio was significantly associated with 1-year eGFR, 1- and 5-year graft survival… Donor recipient size-mismatch, measured by BSA-ratio, is a critical factor influencing post-transplant outcome"*
**Introduction**				
Background/rationale	2	Explain the scientific background and rationale for the investigation being reported	5	*"Kidney transplantation offers significant advantages… Optimal donor-recipient matching is essential… donor kidney should be able to provide sufficient nephron mass to match the recipient's demands… current literature is conflicting..."*
Objectives	3	State specific objectives, including any prespecified hypotheses	6	*"In this study, we analyzed various donor-recipient size mismatch measures and their impact on kidney transplant outcomes."*
**Methods**				

Study design	4	Present key elements of study design early in the paper	7-11	*"We performed a population-cohort study using data from the Organ Procurement & Transplantation Network (OPTN) registry…"*
Setting	5	Describe the setting, locations, and relevant dates, including periods of recruitment, exposure, follow-up, and data collection	7	*"We included adult recipients… between January 1, 2010, and June 30, 2024… Data were extracted on June 30, 2025…"*
Participants	6	(*a*) *Cohort study*—Give the eligibility criteria, and the sources and methods of selection of participants. Describe methods of follow-up<br>*Case-control study*—Give the eligibility criteria, and the sources and methods of case ascertainment and control selection. Give the rationale for the choice of cases and controls<br>*Cross-sectional study*—Give the eligibility criteria, and the sources and methods of selection of participants	7	*"Exclusion criteria were uncontrolled DCD, donors aged under 18, multi-organ… retransplants, en-bloc paediatric kidney transplants, retransplants and missing data for donor-recipient BSA ratio…"*
		(*b*) *Cohort study*—For matched studies, give matching criteria and number of exposed and unexposed<br>*Case-control study*—For matched studies, give matching criteria and the number of controls per case	Not applicable (no matched design)	
Variables	7	Clearly define all outcomes, exposures, predictors, potential confounders, and effect modifiers. Give diagnostic criteria, if applicable	7-9	*"The primary exposure was donor-recipient body surface area (BSA) ratio, defined as donor BSA divided by recipient BSA. BSA was calculated using du Bois formula, based on height and weight at the time of donation or transplant… Primary outcome of interest was recipient 1-year estimated*

				glomerular filtration rate (eGFR, using CKD-EPI 2021 formula), with recipients losing their graft before 1 year given a nominal eGFR value of 10 ml/min/1.73m2"
Data sources/ measurement	8*	For each variable of interest, give sources of data and details of methods of assessment (measurement). Describe comparability of assessment methods if there is more than one group	7-9	*"Data from the Organ Procurement & Transplantation Network (OPTN) registry… BSA was calculated using du Bois formula…"*
Bias	9	Describe any efforts to address potential sources of bias	9-10	*"Multiple imputation was to account for missing data… Multivariable linear regression model was used to analyze the impact of donor-recipient size mismatch… Potential confounders were selected based on previous research and clinical expertise… We employed hierarchical modelling to adjust for transplant center…"*
Study size	10	Explain how the study size was arrived at	12	*"We included 156,917 kidney recipients… Information on cohort selection is given in our study flow diagram (Figure 1)."*

Continued on next page

Quantitative variables	11	Explain how quantitative variables were handled in the analyses. If applicable, describe which groupings were chosen and why	10-11	*"Restricted cubic splines (RCS) with 4 knots… were used to analyze continuous variables… Continuous variables are given as median and interquartile range"*
Statistical methods	12	(*a*) Describe all statistical methods, including those used to control for confounding	9-11	*"Multivariable linear regression… Multivariable Cox regression… Multivariable logistic regression…"*
		(*b*) Describe any methods used to examine subgroups and interactions	10	*"Additional models were built allowing for interactions, to assess whether the various donor-recipient mismatch exposures were inaccurate based on specific donor, recipient or transplant factors"*
		(*c*) Explain how missing data were addressed	9	"*Multiple imputation was performed by generating 20 imputed datasets to account for missing data, using predictive mean matching with bootstraps to build rich additive restricted cubic spline models (aregImpute; Hmisc R package). This approach preserves non-linear relationships. In the imputation models, we included outcome variables to preserve their relationship with missing covariates. For time-to-event outcomes, we included the event indicator variable and cumulative hazard of the event"*

		(*d*) *Cohort study*—If applicable, explain how loss to follow-up was addressed<br>*Case-control study*—If applicable, explain how matching of cases and controls was addressed<br>*Cross-sectional study*—If applicable, describe analytical methods taking account of sampling strategy	7	*"Data were extracted… ensuring all patients had a minimum of 1 year post transplant follow-up period"*
		(*e*) Describe any sensitivity analyses	15	*"Sensitivity analysis was performed to account for variation seen between transplant centers"*
**Results**				
Participants	13*	(a) Report numbers of individuals at each stage of study—eg numbers potentially eligible, examined for eligibility, confirmed eligible, included in the study, completing follow-up, and analysed	12	*"We included 156,917 kidney recipients from January 1, 2010, to June 30, 2024… Information on cohort selection is given in our study flow diagram (Figure 1)."*
		(b) Give reasons for non-participation at each stage	7	"*Exclusion criteria were uncontrolled DCD, donors aged under 18, multi-organ… retransplants, en-bloc paediatric kidney transplants, retransplants and missing data for donor-recipient BSA ratio… Information on cohort selection is given in our study flow diagram (Figure 1).*"
		(c) Consider use of a flow diagram	27	*Figure 1*
Descriptive data	14*	(a) Give characteristics of study participants (eg demographic, clinical, social) and information on exposures and potential confounders	12, 28	*"Key cohort demographics are given in Table 1, with additional demographics and description of missing data in Supplementary Table 1"*
		(b) Indicate number of participants with missing data for each variable of interest	28	*Table 1 and Supplementary Table 1*

		(c) *Cohort study*—Summarise follow-up time (eg, average and total amount)	7	*"…all patients had a minimum of 1 year post transplant follow-up period"*
Outcome data	15*	*Cohort study*—Report numbers of outcome events or summary measures over time		
		Case-control study—Report numbers in each exposure category, or summary measures of exposure		
		Cross-sectional study—Report numbers of outcome events or summary measures	-	*In table legends of all model summary tables*
Main results	16	(*a*) Give unadjusted estimates and, if applicable, confounder-adjusted estimates and their precision (eg, 95% confidence interval). Make clear which confounders were adjusted for and why they were included	12-18	"*A multivariable linear regression model was used… adjusting for a wide range of factors… full model details in Supplementary Table 3…"*
		(*b*) Report category boundaries when continuous variables were categorized	10	*"Restricted cubic splines (RCS) with 4 knots were used to analyze continuous variables to avoid the assumption of non-linear relationships"*
		(*c*) If relevant, consider translating estimates of relative risk into absolute risk for a meaningful time period		

Continued on next page

Other analyses	17	Report other analyses done—eg analyses of subgroups and interactions, and sensitivity analyses	14-15	*"interaction between donor-recipient BSA ratio and donor BMI… donor age… donor-recipient sex mismatch… recipient age. Sensitivity analysis was performed to account for variation seen between transplant centers"*
**Discussion**				
Key results	18	Summarise key results with reference to study objectives	18	*"We demonstrated that donor-recipient BSA ratio is a critical factor influencing post-transplant outcome, with kidneys from smaller donors into larger recipients consistently showing worse outcome"*
Limitations	19	Discuss limitations of the study, taking into account sources of potential bias or imprecision. Discuss both direction and magnitude of any potential bias	20-21	"*One of the limitations of this study is that donor and recipient BSA were used as surrogates for nephron mass and nephron demand respectively… Furthermore, BSA was calculated from height and weight using the Du Bois formula, which provides an estimate rather than a precise measurement… Another limitation is the retrospective design of the study and potential for selection bias… more formal simulation studies are needed to explore this…"*
Interpretation	20	Give a cautious overall interpretation of results considering objectives, limitations, multiplicity of analyses, results from similar studies, and other relevant evidence	18-22	*Discussion*
Generalisability	21	Discuss the generalisability (external validity) of the study results	22	*"These findings support incorporating donor-recipient BSA ratio into offering schemes to optimize kidney offering, and*

				including it as a potential critical confounder in both prospective clinical trials and observational studies"
**Other information**				
Funding	22	Give the source of funding and the role of the funders for the present study and, if applicable, for the original study on which the present article is based	26	*"SJT was funded for this work via a Medical Research Council Clinical Research Training Fellowship (MRC/Y000676/1), which was part funded by Kidney Research UK. This study is funded by the National Institute for Health and Care Research (NIHR) Blood and Transplant Research Unit in Organ Donation and Transplantation (NIHR203332), a partnership between NHS Blood and Transplant, University of Cambridge and Newcastle University. The views expressed are those of the author(s) and not necessarily those of the NIHR, NHS Blood and Transplant or the Department of Health and Social Care."*

*Give information separately for cases and controls in case-control studies and, if applicable, for exposed and unexposed groups in cohort and cross-sectional studies.

Note: An Explanation and Elaboration article discusses each checklist item and gives methodological background and published examples of transparent reporting. The STROBE checklist is best used in conjunction with this article (freely available on the Web sites of PLoS Medicine at http://www.plosmedicine.org/, Annals of Internal Medicine at http://www.annals.org/, and Epidemiology at http://www.epidem.com/). Information on the STROBE Initiative is available at www.strobe-statement.org.
